# *De novo* protein identification in mammalian sperm using *in situ* cryo-electron tomography and AlphaFold2 docking

**DOI:** 10.1016/j.cell.2023.09.017

**Published:** 2023-10-20

**Authors:** Zhen Chen, Momoko Shiozaki, Kelsey M. Haas, Will M. Skinner, Shumei Zhao, Caiying Guo, Benjamin J. Polacco, Zhiheng Yu, Nevan J. Krogan, Polina V. Lishko, Robyn M. Kaake, Ronald D. Vale, David A. Agard

**Affiliations:** 1. Department of Biochemistry and Biophysics, University of California, San Francisco, San Francisco, CA, USA.; 2. Department of Cellular and Molecular Pharmacology, University of California, San Francisco, San Francisco, CA, USA.; 3. Janelia Research Campus, Howard Hughes Medical Institute, Ashburn, VA, USA.; 4. J. David Gladstone Institutes, San Francisco, CA, USA.; 5. Quantitative Biosciences Institute (QBI), University of California San Francisco, San Francisco, CA, USA.; 6. Department of Molecular and Cell Biology, University of California, Berkeley, CA, USA.; 7. New address: Department of Cell Biology and Physiology, Washington University in St. Louis, School of Medicine, St. Louis, MO, USA.; 8. Lead Contact

## Abstract

To understand molecular mechanisms of cellular pathways, contemporary workflows typically require multiple techniques to identify proteins, track their localization, and determine their structures *in vitro*. Here, we combined cellular cryo-electron tomography (cryoET) and AlphaFold2 modeling to address these questions and understand how mammalian sperm are built *in situ*. Our cellular cryoET and subtomogram averaging provided 6.0 Å reconstructions of axonemal microtubule structures. The well-resolved tertiary structures allowed us to unbiasedly match sperm-specific densities with 21,615 AlphaFold2-predicted protein models of the mouse proteome. We identified Tektin 5, CCDC105 and SPACA9 as novel microtubule-associated proteins. These proteins form an extensive interaction network crosslinking the lumen of axonemal doublet microtubules, indicating their roles in modulating the mechanical properties of the filaments. Indeed, *Tekt5* −/− sperm possess more deformed flagella with 180° bends. Together, our studies presented a cellular visual proteomics workflow and shed light on the *in vivo* functions of Tektin 5.

## eTOC blurb

Chen et al. reported reconstruction of microtubule doublets in mammalian sperm using *in situ* cryo-electron tomography. Unbiased matching of sperm-specific structures to the AlphaFold2 library allowed *de novo* protein identification of microtubule inner proteins. A transgenic mouse model revealed the functional importance and partial redundance of Tektin 5 in sperm.

## Graphical Abstract

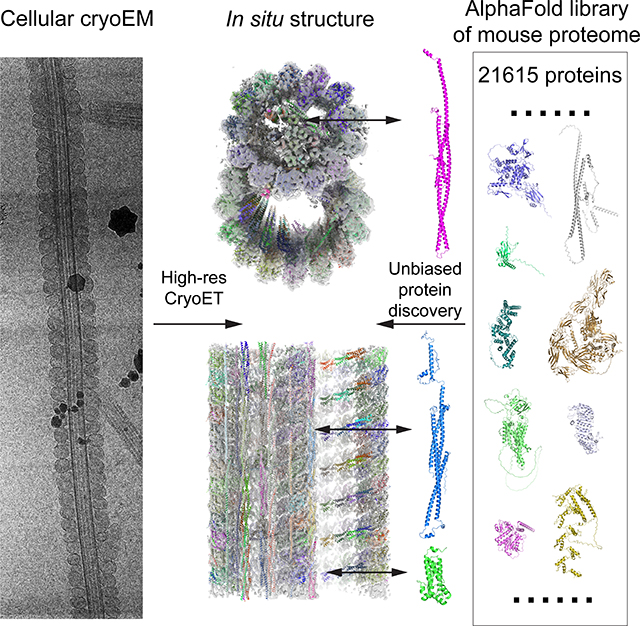

## INTRODUCTION

Natural fertilization requires the rhythmic beating motion of sperm flagella to propel the cell toward the egg ^1,2^. This coordinated and repetitive bending of sperm flagella relies on macromolecular machinery to generate periodic force and endure mechanical stresses. Genetic analyses of infertility have so far offered only an incomplete list of protein candidates in sperm ^1^. Additionally, we currently lack high-resolution information of sperm macromolecular complexes to understand their assemblies and functions at the molecular level.

Eukaryotic motile cilia and flagella share a conserved filamentous structure, the axoneme that has an overall architecture of nine doublet microtubules (doublets) surrounding two singlet microtubules ^3–6^. These cytoskeletal filaments are extensively decorated externally and internally by proteins required for the various beating motions and the structural integrity of flagella ^3,4^. Notably, sperm from different species can differ substantially in their morphologies, functions (*e.g.*, swimming behaviors), and genetics ^7–9^. In particular, mammalian sperm flagella are much longer, wider, and must withstand larger bending torques compared to other motile cilia ^10,11^. Despite their crucial roles of sperm axonemes in fertility and speciation, our understanding of their unique adaptations remains limited.

Isolation of axonemal complexes from non-sperm motile cilia combined with single-particle cryoEM analyses have provided high-resolution reconstructions (better than 4 Å) ^12–16^. The rich structural information on the tertiary folds and side chains has allowed confident assignments of protein identities in the EM reconstructions. However, careful optimization of purification strategies is required to avoid partial loss of components ^14,16,17^. Thus, the truly intact structures are not guaranteed by the end of purification in general. On the other hand, direct visualization of macromolecular complexes in mammalian sperm using cryogenic focused ion beam-scanning electron microscopy (cryoFIB-SEM) and *in situ* cryo-electron tomography (cryoET) indicated that there are indeed mammalian sperm-specific features ^18–20^. However, the current cryoET subtomogram averaging of axonemal microtubule structures is limited to ~10–20 Å resolutions, mainly due to alignment inaccuracies. At such resolutions, tertiary structures of the proteins are rarely resolved and multi-protein complexes appear as blobs, making it challenging to determine the identities of individual sperm proteins.

Here, our *in situ* cryoET and subtomogram averaging has achieved up to 6.0 Å reconstructions of native microtubule structures in mouse and human sperm samples. The well-resolved tertiary structures in our cryoEM maps allowed us to survey the 21,615 AlphaFold2-predicted protein models of the mouse proteome and unbiasedly identify matching ones. Such a visual proteomics approach helped us to discover novel microtubule-associated proteins in mammalian sperm, localize them in cells, and determine their native structures and interaction network without cell disruption and biochemical purification. We also generated CRISPR-knockout mouse lines and showed that the newly identified Tektin 5 is important for the structural integrity of the flagella. Our studies established a cellular visual proteomics workflow and provided the structural and functional basis of sperm proteins in the microtubules.

## RESULTS

### *In situ* structures of sperm doublets at subnanometer resolutions

**Figure 1.**
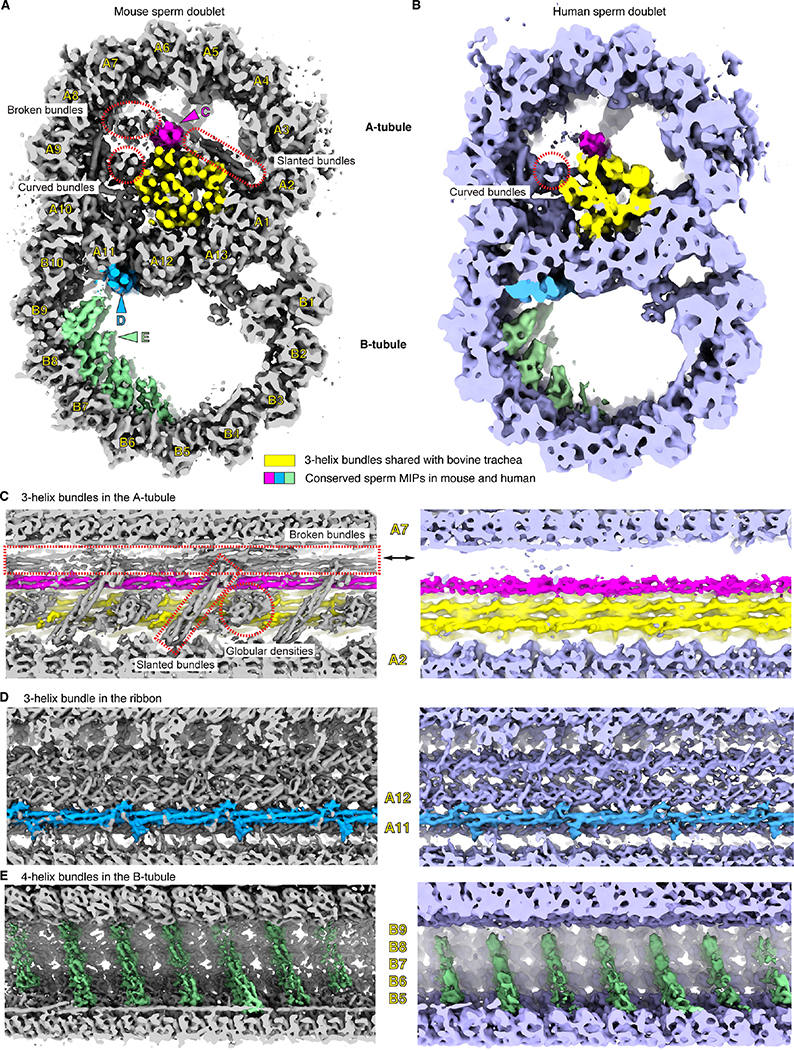
The 3D reconstructions of mouse and human sperm doublets revealed novel MIPs. (**A**), (**B**) Transverse cross-section views of the doublets of mouse (**A**) and human (**B**) sperm. Conserved sperm MIP densities are highlighted (pink, blue and green) and the corresponding viewing angles of (**C**)-(**E**) are indicated (colored arrowheads). The 3-helix densities in A-tubule shared with Bovine trachea doublets (EMD-24664) are colored (yellow) ^13^. Divergent sperm densities are also indicated (red dashed shapes). Individual protofilaments of the doublets are labeled as A1–13 and B1–10. (**C**)-(**E**). Zoom-in views of the conserved sperm MIP densities along the longitudinal axis. In (**C**), mouse sperm-specific densities are indicated and labeled (red dashed shapes, see more in Figures S2 and S3). In (**E**), although the striations are 8 nm apart from one another, the overall periodicity is 48 nm.

Freshly extracted mouse sperm were treated with the dynein inhibitor EHNA (erythro-9-(2-hydroxy-3-nonyl)adenine; 10 mM), which immediately stopped the beating motion of sperm flagella ^21^. Subsequently, the inhibited sperm were vitrified on EM grids. To facilitate cryoET imaging which is limited by sample thickness, lamellae of ~300 nm-thickness were generated by cryo FIB-SEM milling. Tilt series were recorded using a 300 kV Krios cryo-electron microscope (cryoTEM) and a dose-symmetric scheme with a tilting increment of 4°. The 4° tilt increment, instead of the commonly used 1–3° ^18,19,22,23^, was used to improve the signal-to-noise ratio of each tilt image while keeping the same angular range and total dose (Figure S1). Three-dimensional classification and refinement of subtomograms corresponding to the 96 nm-repeating units were performed as reported previously ^19^. We then performed local refinement on the 48 nm-repeating structures of doublets focusing on the microtubules, aiming to reach the highest possible resolution (see the workflow shown in Figure S1). The newly developed RELION4 was used to refine the 3D reconstructions and achieved 7.7 Å overall resolution (FSC = 0.143) (Figures 1A and S2A–B, see Methods) ^24^. The improvement of resolution compared to RELION3 comes from more accurate CTF estimation and alignment of tilt series as these parameters of each tilt image were iteratively refined relative to the 3D reconstructions (Figure S2A) ^24^. Densities of microtubule inner proteins (MIPs) from mouse axonemes that repeat every 16 nm were observed despite of the overall periodicity of 48 nm (Figures 1C to E). In addition, we re-processed a previous human sperm dataset and achieved 10.3 Å for the 48 nm-repeating structures of the doublets (FSC = 0.143) (Figures 1B and S3A–B) ^19^. Individual α-helices for the tubulins and MIPs are resolved in both maps. These maps were then compared to the published cryoEM map of isolated doublets from bovine trachea reconstructed by single-particle cryoEM ^13^, which were low-pass filtered to comparable resolutions of 7.5 Å and 10 Å, respectively (Figures S2C and S3C). Similar levels of detail in secondary and tertiary structures were resolved, validating the resolution estimates of our 3D reconstructions. To our knowledge, these resolutions are currently the highest achieved by cryoET for any *in situ* axonemal structures (12 Å maps were reported previously for equivalent structures from *Tetrahymena* cilia ^23,25^).

Our 3D reconstructions of mouse and human sperm doublets reveal densities similar to the ones from bovine trachea doublets ^13^, as well as sperm-specific densities (colored densities in Figure 1). Inside the A-tubule of mouse sperm axonemes, twelve helical bundles form a filamentous core parallel to the longitudinal axis, whereas only eight helical bundles, identified as Tektins 1–4, are present in bovine trachea cilia (Figures 1A and S2D) ^13^. Among the four mouse sperm-specific helical bundles, only one is a continuous 3-helix bundle that runs along the entire length of the doublets (Figures 1A and 1C). This continuous bundle is also found in human sperm doublets (Figures 1A–C). The other three bundles, the two broken bundles and the curved bundles, all have breaks within the 48-nm periodic structure and appear different in mouse and human sperm (Figures. S2D and S3D–G). In particular, the two broken straight 3-helix bundles have very low occupancy in the human sperm doublet (Figures 1B and S3D–E), while one of the curved helical bundles is connected to the microtubule lumen in human but not mouse sperm (Figures S2D and S3F–G). In the mouse sperm doublet, we also observed unique “oblique” helical densities oriented ~45° relative to the filament axis and a globular domain next to it every 16 nm (Figure 1C). These comparisons indicate there are sperm-specific MIPs compared to mammalian trachea cilia and also diversifications among mammalian sperm in the A-tubule of the doublets.

Outside the A-tubule, we found novel densities that are conserved in both mouse and human sperm doublets. A continuous three-helix bundle with multiple protrusions is situated at the external interface between A11 and A12 protofilaments, previously named the “ribbon” of doublets (Figure 1A, 1B and 1D) ^26^. Inside the B-tubule, there are groups of four-helix bundles lining the inner surface of tubulins from the B4-B9 protofilaments. These four-helix bundles are stacked next to one another along the helical pitch of the microtubule, consistent with the previously reported striation density at lower resolutions (Figure 1E) ^19^. Together, these data reveal that the mammalian sperm doublets have the most extensive MIP network of any microtubule structure observed to date. While the B-tubule appears similar, mouse sperm have more MIPs than human sperm in the A-tubule.

### *De novo* protein identification using AlphaFold2

The clearly resolved secondary and tertiary structures allowed us to interpret maps and build pseudo-atomic models. First, we were able to identify densities corresponding to the 29 MIPs observed in bovine trachea cilia (Figure S4) ^13^, suggesting their orthologs or homologs are likely present in sperm axonemes. We then sought to identify proteins contributing to the conserved sperm densities in mouse and human doublets (highlighted in Figure 1C–E). Since most of these features repeat every 16 nm along the axoneme axis, we performed focused refinement of the 16-nm repeating structures in the A- and B-tubules of mouse doublets separately and the larger number of subtomograms further improved the resolutions of averages to 6.0 Å and 6.7 Å, respectively (Figure S5).

**Figure 2.**
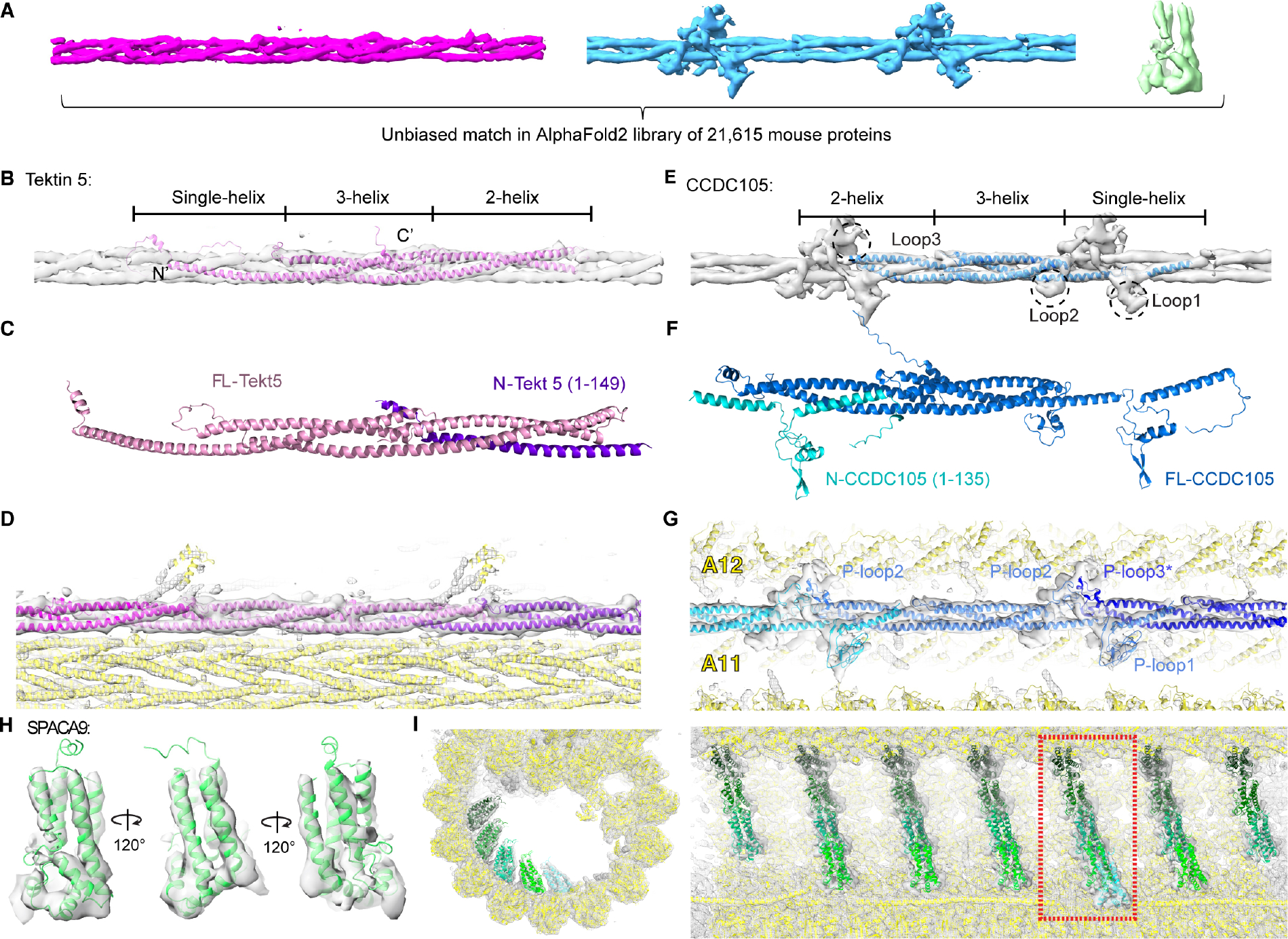
*De novo* protein identification of sperm MIPs assisted by AlphaFold2. (**A**) Conserved densities in mouse and human sperm were segmented from the averages of 16-nm repeats of mouse sperm doublets and searched in the AlphaFold2 library of the mouse proteome (21,615 proteins). (**B**) The predicted structure of Tektin 5 based on AlphaFold2 was fitted into the continuous 3-helix bundle. (**C**) Modeling of a complex formed by a full-length Tektin 5 and a truncated one (N-Tekt 5: a.a. 1–149) using Colabfold ^35^. (**D**) Fitting and modeling of Tektin 5s into the 3-helix bundle densities in the A-tubule. The nearby densities accounted for by other proteins are also shown (yellow ribbon). (**E**) An unbiased search in the AlphaFold2 library identified CCDC105 as the candidate for the continuous 3-helix density at the ribbon. The three conserved proline-rich loops among CCDC105 orthologs could account for the protrusion densities but were not modeled (See Figures S6C–D). (**F**) Modeling of a complex formed by a full-length CCDC105 and a truncated one (N-CCDC105: a.a. 1–135) using Colabfold ^35^. (**G**) Fitting and modeling of CCDC105 into the 3-helix bundle density at the ribbon. The nearby densities are accounted for by other proteins (yellow ribbon). (**H**) The AlphaFold2 model for SPACA9 was directly fitted into the density and viewed from different angles. (**I**) Two orthogonal views of the striations of SPAC9 in the B-tubule. Different SPACA9 molecules are colored with different shades of green. The left panel showed a particular striation indicated in the right panel (the dashed rectangle).

We then aimed to develop a general strategy to assign or narrow down the protein identities of the densities in the 6–7 Å reconstructions. Unassigned densities from our maps were manually isolated and unbiasedly matched to the predicted tertiary structures from the AlphaFold2 mouse proteome library (21,615 proteins) using the COLORES program from the SITUS package (Figure 2A) ^27,28^. The best poses for 21,615 mouse proteins were scored and ranked by the cross-correlation scores calculated by COLORES ^28^. We envisioned that AlphaFold2 may not be able to predict the inter-domain orientations of multi-domain proteins with no or limited inter-domain contacts. Thus, we tested this workflow using densities corresponding to known single-domain MIPs (CFAP20 and PACRG) and a multi-domain MIP (NME7) as controls. The correct PDBs corresponding to the selected densities all came up as top hits using this unbiased proteome-wide search (Data S1), indicating this visual proteomic approach can reliably identify proteins with matching tertiary structures.

For the continuous 3-helix densities in the A-tubule, the best hit was Tektin 5 (Figure S6A), a Tektin found only in the mammalian testis and sperm in previous proteomic studies (Figures 2B and S6A–B, see methods for details) ^29–31^. Although Tektin 5 has no reported structure, AlphaFold2 predicted that it possesses single-helix, 3-helix and 2-helix segments from the N- to C-termini (Figure 2B) ^27^, a tertiary structure that is almost identical to the ones reported for Tektin 1–4 in bovine trachea cilia ^13^. The ColabFold, an AlphaFold2-based Google notebook, was then used to model how two copies of Tektin 5 molecules interact ^32^. The resulting complexes suggest the single-helix N-terminal region of one Tektin 5 could interact with the 2-helix C-termini of the other molecule (Figure 2C), indicating its potential to self-polymerize and form a quasi-continuous 3-helix bundle. Indeed, multiple Tektin 5 could be fitted into the continuous 3-helix densities with 16-nm periodicity, with minor adjustments of the orientations of individual α-helices of the original AlphaFold2 model (Figure 2D). Upon manual inspection of the hit list, Tektin 1–4 were also among the top 10 hits (Figures S6A and S6B); this finding corroborated the robustness of our search method in finding proteins with matching tertiary structures. We assigned these densities as Tektin 5 since it is uniquely present in mammalian sperm based on previous proteomic studies ^30,31^ and such densities are absent in bovine trachea cilia that only contain Tektin 1–4 ^13^.

For the continuous 3-helix densities with protrusions at the ribbon, CCDC105 (coiled-coil domain containing protein 105) was identified in the top 20 hits from the unbiased search in AlphaFold2 mouse proteome library, along with Tektin 1–5 (see Data S2 for the top 30 hits). Previous proteomic studies revealed that CCDC105 was found in mammalian sperm and the testis but not other tissues ^29–31^. CCDC105 adopts a similar overall tertiary structure as Tektins 1–5 based on AlphaFold2 prediction (Figure 2E) ^27^, suggesting it is a yet uncharacterized Tektin homolog. However, there are three proline-rich loops in CCDC105 that are uniquely conserved across CCDC105 orthologs (Figures 2E, S6C and S6D) and they likely form structured loops like the ones observed in other axonemal complexes ^33,34^. We also modeled how two copies of CCDC105 would interact using AlphaFold2/ColabFold ^35^. The predicted interface again involves coiled-coil interactions between the single-helix segment of one CCDC105 and the two-helix segment of the other (Figure 2F). Furthermore, CCDC105 fits well into the continuous 3-helix densities at the ribbon, with their characteristic proline-rich loops matching the protrusions in our density maps (Figure 2G). Notably, we could not swap the fitting of Tektin 5 and CCDC105 into these two 3-helix bundles after extensive trials, mostly due to the different orientations and lengths of the α-helices (Figure S6F).

We also extracted the 4-helix bundles at the B-tubule striations and performed an unbiased search against the AlphaFold2 library. SPACA9 (Sperm Acrosome-Associated Protein 9) was found to be the best hit (Figure 2H and 2I, see Data S3 for the top 30 hits). SPACA9 was previously found in various ciliated organs in humans (testis, fallopian tubes and lung) ^29^ and its tertiary fold is so unique that no other homologous protein was found in the top 30 ranked structures from the unbiased search. Interestingly, no match was found when the search was done against the CATH library that curates non-redundant domains of published PDBs ^36^. Thus, the capability of AlphaFold2 to predict protein structures accurately, especially for the ones without published homologous structures, is critical to carrying out the unbiased proteome-wide survey.

**Figure 3.**
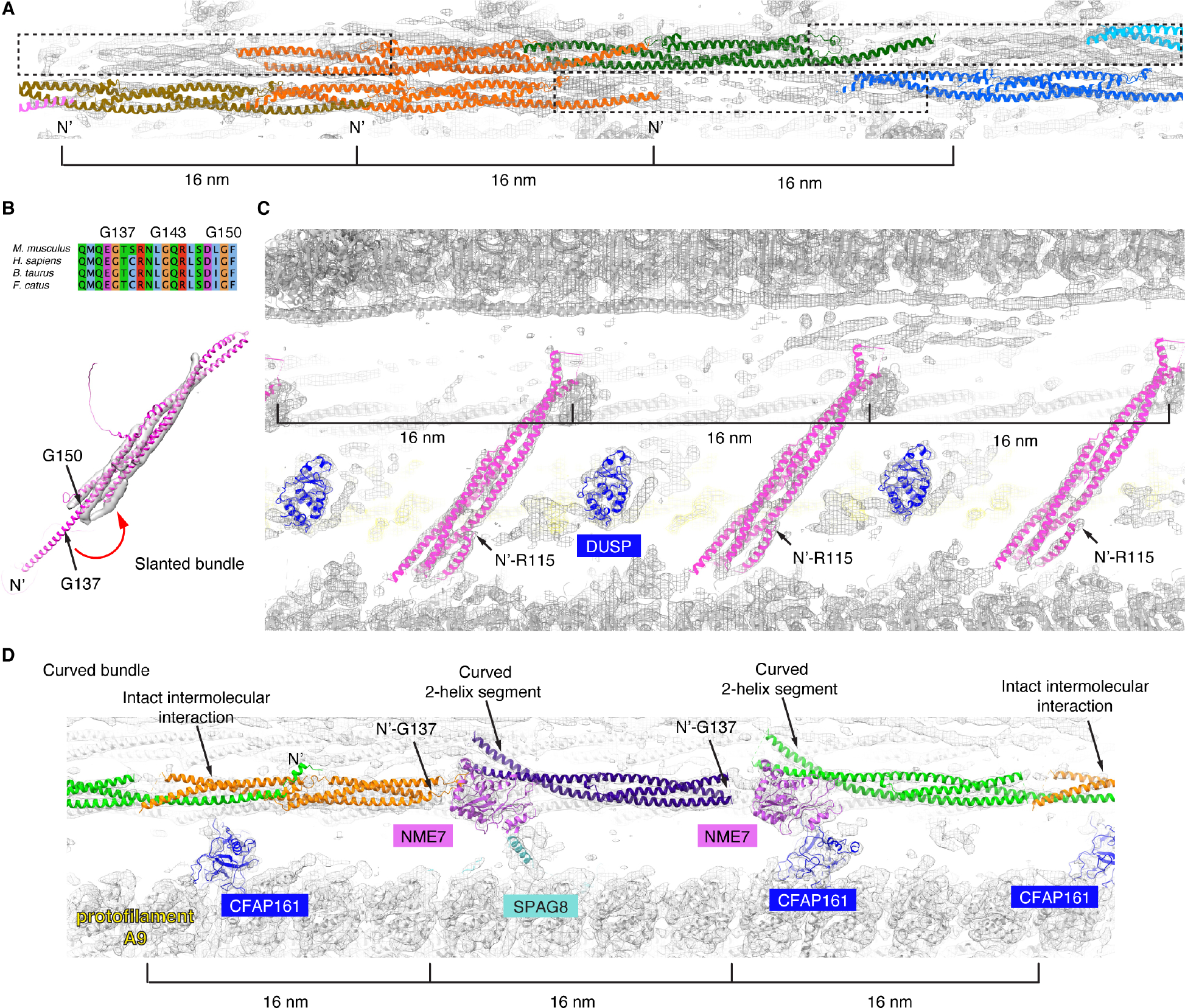
Conformational plasticity of Tektin 5. (**A**) The two broken 3-helix bundles could be explained by two complete and a third partial copies of Tektin 5 (dashed rectangles) per 48-nm repeat, instead of three Tektins in the continuous 3-helix bundle. (**B**) The AlphaFold2 model of mouse Tektin 5 was fitted into the slanted helical densities. Sequence alignment of Tektin 5 from *M. musculus*, *H. sapiens*, *B. taurus* and *F. catus* is shown from Q133-F151 (the numbering of amino acids is based on *M. musculus* Tektin 5). The conserved Gly137, Gly143 and Gly150 are near the turning point of the bent α-helix. (**C**) The fitting of Tektin 5 and DUSP3 protein (its homologs are also possible candidates) into the 16-nm repeating features, see the same view of the map in Figure 1C. (**D**) Three modified Tektin 5 were fitted into the densities of curved bundles in the mouse sperm doublet (as indicated in Figure 1A). The intact intermolecular interaction interface, N-termini of the Tektin 5s and curved 2-helix segments are indicated (arrows). Nearby MIPs shared between mouse sperm flagella and bovine trachea cilia are also colored and labeled (NME7, CFAP161, SPAG8). (**E**) The cross-section schematic is shown. The highlighted models of panels A-D are indicated using arrows.

We next focused on the mouse sperm-specific densities. There are two 3-helix bundles that appear to be similar to the ones formed by Tektins 1–5, apart from the discontinuous sections (Figures 3A and S2D). We also applied the unbiased search method to the slanted and curved helical bundles (Figures 1C and S2D). Intriguingly, Tektin 1–5 and CCDC105 were found to be among the top 30 hits in both cases while no other PDB among the top 200 fits better, albeit only parts of the structures are observed for the densities (see Data S4). Other hits among the top 200 do not match the secondary structures of the target densities upon visual inspections, suggesting Tektin 1–5 and CCDC105 are the only proteins in mouse proteome that adopt such conformations. For the slanted helical densities, there is an additional α-helix connecting to the position where the missing single helix was expected to originate and is folded back by ~180° (Figures 3B and 3C). Interestingly, Tektin 5, but not Tektin 1–4, has multiple conserved Gly residues among its orthologs at this turning region, making it plausible that Tektin 5 could adopt the bent-helix conformation. At a lower threshold, this bent helix is connected to a nearby globular domain that also repeats every 16 nm (Figure S7A). The unbiased proteome-wide search suggests this globular domain matches the tertiary structure of multiple DUSP proteins (Dual Specificity Phosphatase 3, 13, 14, 18, 21 and 29) (Figures 3C and S7B).

**Figure 4.**
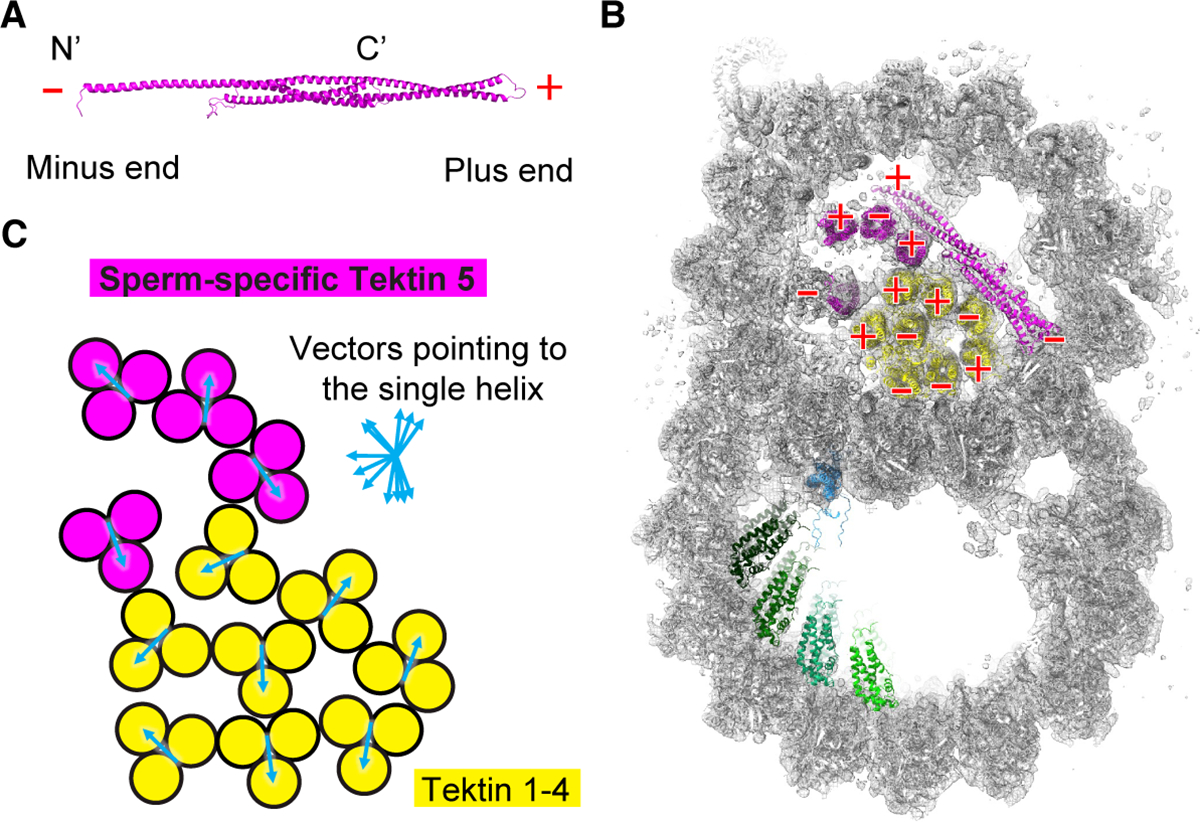
Sperm doublets are composed of microtubules and extensive coiled-coil bundles. (**A**) The plus and minus ends of Tektin 5 were named based on the N- and C-termini of the protein. (**B**) The cross-section view of the mouse sperm doublets shows the polarities of 3-helix bundles pointing toward the readers. (**C**) The orientations for each 3-helix bundle were represented by a vector starting from the middle point of the 2-helix segment and pointing toward the single-helix segment of the other Tektin molecule.

For the curved helical bundles, there are three 16-nm groups of densities within every 48-nm repeat (Figures 3D and S2D). These densities can be explained by three modified Tektin 5 molecules, in which the two intermolecular interfaces near the two NME7s (a previously known MIP shared with bovine trachea cilia) are disrupted (Figure 3D). The first and second Tektin 5s lack densities for the single-helix segment beyond the conserved Gly137 (mouse), while the second and third Tektin 5s possess curved 2-helix segments (Figure 3D). Both modifications of Tektin 5s are necessary to avoid direct steric clashes with the two NME7s, which adopt similar conformations in bovine trachea and mouse sperm doublets. As curved bundles were not observed in the bovine trachea cilia that only contain Tektins 1–4 ^13^, we hypothesize that Tektin 5 has evolved to adopt multiple conformations and positions within sperm axonemes (Figure 4E).

In summary, the various helical conformers of Tektin 5, together with the more uniform 3-helix bundles of Tektin 1–4, are arranged with different polarities (Figures 4A and 4B) and orientations (Figure 4C), forming the most extensive MIP network inside microtubules discovered to date.

### Additional validation and redundancy of Tektin5

To further validate our *de novo* protein assignments, we used mass spectrometry-based proteomics. Mouse sperm were isolated and extracted using salt buffers with increasing denaturing capabilities (E1: 0.1% Triton, E2: NaCl, E3: KSCN, E4: Urea, and E5: 10% SDS) and the extractions were analyzed using SDS-PAGE and western blotting (Figure S8A). α-Tubulins could be detected in KSCN and Urea extractions but not in others (Figure S8B), suggesting the microtubule doublets are disassembled and the MIP candidates are likely present in these two extractions. After analyzing the E1-E5 fractions by MS, proteins with significant changes in abundance between fractions were clustered into six distinct groups based on the correlation of intensity profile (Figures S8C and S8D and Tables S1 and S2) ^37,38^. Gene ontology (GO) analyses suggest that cluster 4 is enriched for proteins involved in cilium, cilium assembly, cytoskeleton and the axoneme, and shows increased intensities in fraction E3, while cluster 6 is enriched for cilia assembly and shows increased intensities in fraction E4 (Figure S8e and table S3) ^39^. The overall change in protein abundance is consistent with the idea that E3 and E4 buffers extracted microtubule-associated proteins in the axonemes. Indeed, 28 of the 29 previously identified MIPs in bovine trachea doublets were reproducibly identified in all three biological replicates of mouse sperm extractions (table S4). The almost complete list of MIPs highlighted the coverage of our biochemical and MS analyses. Importantly, SPACA9 was reproducibly identified in fraction E3, while Tektin 1–5 and CCDC105 were reproducibly identified in fractions E3 and E4, with high protein intensity and a range of 3 to 34 unique peptide identifications per replicate (Figure S8F, table S4). Only one DUSP protein, DUSP3, was identified in two of three replicates in fraction E4 fraction (Figure S8F, table S4). However, additional analyses are required to identify these proteins. Moreover, AlphaFold2 models for the other candidates from fractions E1–5 were inspected but no additional candidates could explain the various densities of helical bundles described above.

### Tektin 5 strengthens the flagella and is partially redundant

**Figure 5.**
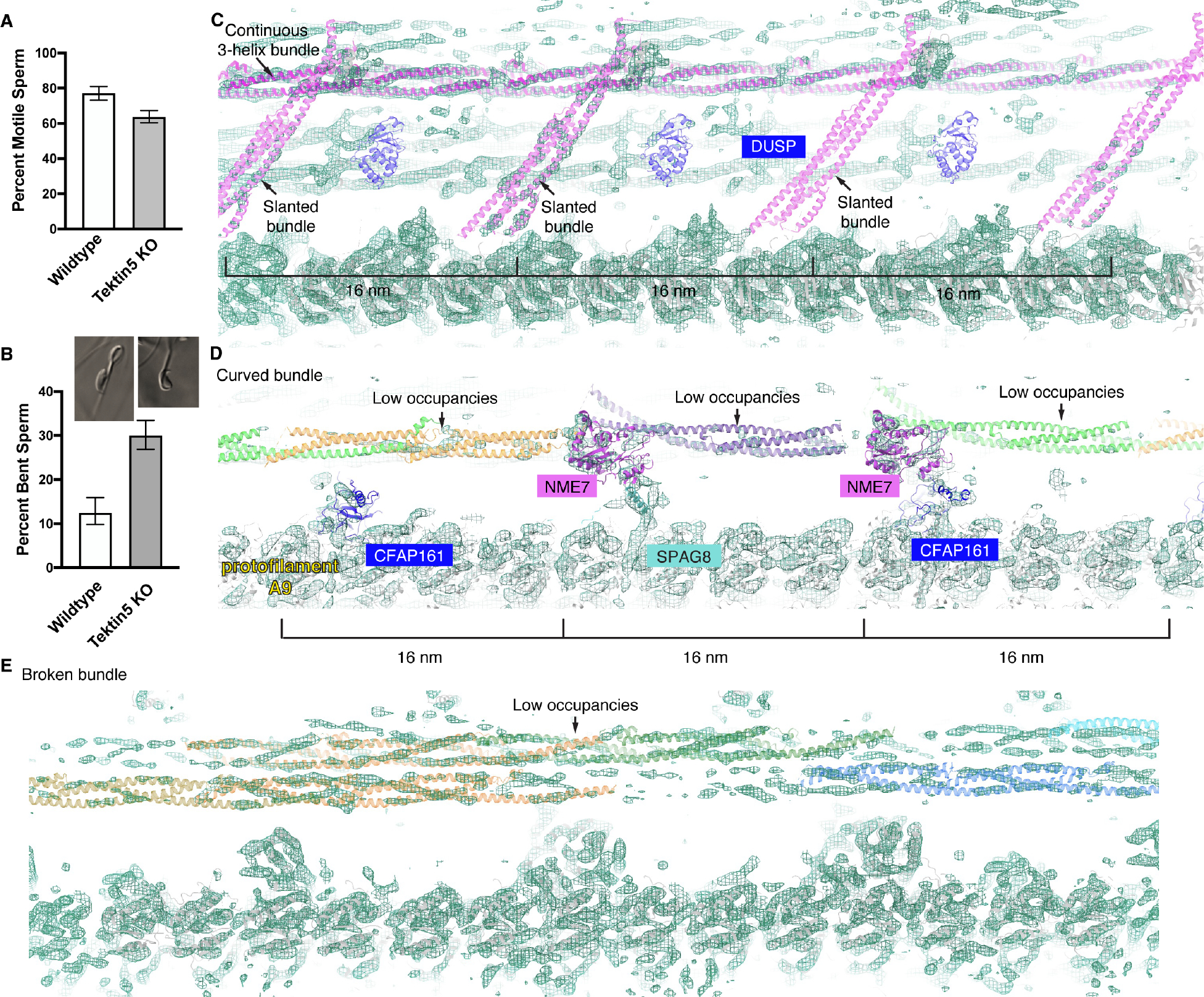
Characterization of mutant *Tekt 5* −/− sperm. (**A**) The percentages of motile sperm from wild-type and *Tekt 5* knockout mice (> 200 cells were counted for each mouse and three knockout −/− mice and two wild-type mice were analyzed, the pool percentage and 95% Confidence Intervals (Wilson/Brown method) were shown). (**B**) The percentages of bent sperm from wild-type and *Tekt 5* knockout mice (> 200 cells were counted for each mouse and three knockout −/− mice and two wild-type mice were analyzed, the pool percentage and 95% Confidence Intervals by Wilson/Brown method were shown). Two examples of bent sperm are shown. (**C**) An overlay of wild-type models with the densities of *Tekt 5* −/− sperm around the slanted bundles. The continuous 3-helix bundle assigned as Tektin 5 (high occupancies) and slanted helical bundles (low occupancies) are shown. The densities corresponding to the DUSP proteins are barely resolved. Note there are substantially less densities for these models compared to Figure 3C. (**D**) An overlay of wildtype models with the densities of *Tekt 5* −/− sperm around the curved bundles. The occupancies of the curved bundles are lower than the other MIPs and tubulins. Note there are substantially less densities for these models compared to Figure 3D. (**E**) The two broken 3-helix bundles have lower occupancies compared to the surrounding MIPs and tubulins. Note there are substantially less densities for these models compared to Figure 3A.

In order to analyze the functions of Tektin 5, we generated knockout mice carrying null alleles of *Tekt5* using CRISPR technologies. To our surprise, the F2 homozygous knockout males are still fertile when they are mated with the WT females (litter size: 7.3 ± 1.4, N=6 mating trials), suggesting some levels of functional redundancy. However, the sperm extracted from the mutant males have a lower fraction of motile cells compared to WT controls (64 ± 3% *vs.* 77 ± 4%) and have a higher percentage of defective flagella with 180° bends (30 ± 3% *vs.* 13 ± 3%) (Figures 5A and 5B), suggesting the mechanical integrity of the cellular structures inside flagella is compromised in the mutant sperm.

We then analyzed the doublets structure of the mutant sperm and compared it to the WT counterpart. We first focused on the various densities that were assigned to be Tektin 5 in both our cellular cryoET (as shown in Figure 3). The continuous 3-helix bundle remains in the mutant (Figure 5C), suggesting a Tektin homolog could substitute on the same position in the absence of Tektin 5. In contrast, the occupancy of densities corresponding to broken and curved helical bundles are much lower compared to the surrounding proteins, such as the tubulins, in the mutant sperm (Figures 5D and 5E), while in wild-type sperm the occupancies are comparable. These comparisons suggest the compensation for the lack of Tektin 5 by other Tektin homologs is low at these sites. For the slanted helical densities that repeat every 16 nm in WT sperm, we observed that two of the slanted helical densities are partially occupied while the last one is almost absent (Figure 5C). Interestingly, the densities corresponding to the DUSP domains next to the slanted helical densities were not resolved, suggesting the lack of Tektin 5 would decrease the recruitment of the neighboring MIP. We did not observe additional differences in densities corresponding to other MIPs. Together, these results suggest that Tektin homologs could partially refill docking sites of Tektin 5 in the mutant sperm.

## DISCUSSION

Our *in situ* cryoET studies have provided high-resolution reconstructions of native structures within mouse and human sperm, enabling us to identify Tektin 5, SPACA9 and CCDC105 as novel components in sperm doublets.

Alignment of gold beads has traditionally been the method of choice to align tomographic tilt series but the positions of gold beads undergo heterogeneous motions due to the sample deformation induced by electrons during cryoET imaging ^40^. The significant improvement in resolutions made possible by RELION4 (Figure S1 and S2A) highlight the benefits of aligning 3D reconstructions of protein complexes with their individual 2D projection views on different tilt images to refine tilt series alignment ^24^. Thus, new methods of aligning the molecular features directly from tilt series while considering their local motions have the potential to further improve the initial alignment of tilt series, and set an even better starting point for subtomogram averaging ^41^.

The application of AlphaFold2 has facilitated structural modeling based on cryoEM reconstructions below 10 Å, where secondary structures are resolved ^42^. However, most studies have focused on protein complexes with known components identified through other approaches such as mass spectrometry analyses of purified complexes. However, this information may not be readily available in other less-studied cell biology processes. Our studies provided the first demonstration that high-resolution cellular cryoET combined with unbiased proteome-wide searches could identify previously unknown components of cellular complexes in their native context. This integrative structural modeling approach offers a powerful alternative to the conventional genetic and cell biology approaches to identify participating protein components and localize them inside cells.

The comparison of MIPs in WT and mutant sperm doublets with bovine tracheal doublets suggest that the assembling of MIPs are modular, and novel MIPs discovered in this study (SPACA9, CCDC105, Tektin 5 and DUSP) are recruited after the commonly shared MIPs bind to the doublets (common MIPs). First, homologs or orthologs of MIPs identified from bovine tracheal doublets are all present in WT and mutant mouse sperm and they adopt similar conformations (Figure S4) ^13^. Second, SPACA9 and CCDC105 crosslink the tubulin dimers only at the exposed lumen sites observed in tracheal doublets, without displacing any common MIPs (Figures 2G, 2I and S4). Interestingly, the remarkable conformational plasticity of Tektin 5 is partially molded by the doublets and common MIPs, as shown by the bent single helix that would otherwise clash with the microtubule wall (Figures 3B and 3C), as well as the missing single helix and curved 2-helix segment that would otherwise clash with NME7, a common MIP (Figure 3D). Lastly, knockout of Tektin 5 decreases the occupancies of the bent Tektin 5 and the nearby DUSP densities (Figure 5C). Such dependency or modularity could potentially be harnessed for modifications during evolution, as we observed that both the bent Tektin 5 and DUSP densities are absent in human sperm doublets.

**Figure 6.**
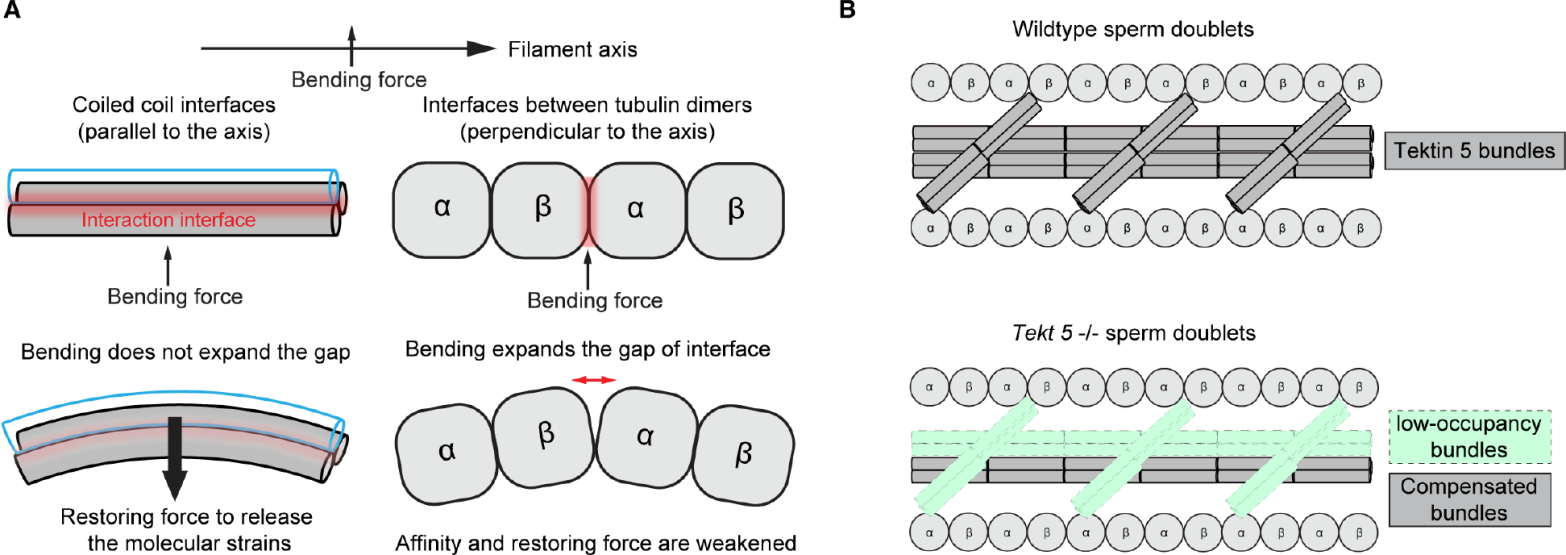
Coiled-coil interfaces are suitable to withstand mechanical stress from orthogonal directions. (**A**) A model of how 3-helix bundles would be able to bear mechanical stress differently compared to the microtubules. The bending curvatures and gaps are exaggerated for illustration purposes. (**B**) A schematic of wild-type and mutant sperm doublets structures highlighting the Tektin 5 bundles and the partial redundancy.

Bending of the axonemes would stress the nine microtubule filaments in nine different directions and mammalian axonemes have to bend to various directions to generate the 3D beating waveforms ^43^. The non-uniform arrangement of helical bundles in sperm doublets could be built to reinforce the doublets to withstand mechanical stress from different directions (Figures 4 and 6). From a structural perspective, the intramolecular and intermolecular coiled-coil interaction interfaces of Tektins are parallel to the microtubule axis so that bending would not expand the gap of these interfaces (Figure 6A). Instead, the bending force would distort the straight helical bundles and the ideal bond angles/lengths. The transition of releasing such molecular strains could provide a restoring force that allows the curved filament to return to the straight conformation. In contrast, the interface between tubulin dimers along the protofilaments is at a plane perpendicular to the filament axis; bending would open the interface and lower the affinity or the potential restoring force. Therefore, the helical bundles are arranged to provide an effective means to bear the bending force, stabilizing the axoneme and flagella.

From a functional perspective, we discovered that mutant mice lacking Tektin 5 have more deformed sperm flagella, yet they remain fertile. This observation aligns with previous studies on knock-out mouse models lacking Tektin-3 or Tektin-4, which also showed deformed sperm but normal fertility ^44,45^, underscoring the general functional redundancy of Tektins. Our structural analyses of *Tekt5* −/− sperm doublets uncovered the compensatory mechanisms of Tektin 5 at the molecular level. Moreover, we discovered that different Tektin 5 conformers were compensated to varying extents, likely due to the differential dependencies of the distinctive interaction interfaces.

The human sperm doublets revealed that the densities formed by Tektin 5 are significantly less resolved compared to the other MIPs (Figure 1), adopt different conformations (Figure S3F), or are completely absent compared to mouse sperm doublets (Figures 1C). This is also consistent with the idea that the bundles formed by Tektin 5 are partially redundant and plastic during evolution so that some extent of degeneration is tolerable. Still, the lower percentages of motile sperm would be selected against in the wild, particularly in species where sperm from multiple males in the female reproductive tracts competing for fertilization.

Our studies combine high-resolution *in situ* cryoET and AlphaFold2 modeling and define a visual proteomic approach of precisely placing proteins in their native cellular environment without the need for labeling, cellular disruption or purification. This workflow has allowed us to uncover the cellular locations and interaction networks of several MIPs, providing insights into how they contribute to the mechanics of flagellar bending. Moreover, this visual proteomic workflow could potentially be applied to other cell biology problems, such as membrane remodeling by viruses and identification of their *in situ* interactors.

### Limitations of the Study

The majorities of the densities in sperm doublets feature well-defined domains that could be isolated and identified using our visual proteomics approach. However, it is possible that there are unknown MIPs that are composed of coiled coils without substantial intramolecular interactions. These proteins may adopt conformations that depend on intermolecular interactions in the context of native complexes, which are not accounted for by AlphaFold2 predictions. Furthermore, map segregation for these types of proteins is challenging at ~6–10 Å resolutions. Improvement of resolution using cellular cryoET is also desirable to resolve the side chain densities in the EM reconstructions and distinguish the proteins with similar tertiary structures. Notably, while this manuscript was under revision, two single-particle cryoEM studies of splayed mammalian sperm doublets were reported ^46,47^. The single-particle cryoEM reconstructions of microtubule doublets from mouse and bovine sperm are indistinguishable from our cryoET reconstruction at our resolutions, suggesting that the sperm doublets are robust enough to withstand gentle biochemical treatments. Importantly, their assignments of protein identity based on side chain densities are consistent with our identification of CCDC105, SPACA9 and the different forms of Tektin 5, validating our cellular visual proteomics approach for *de novo* protein discovery. In the future, a more systematic characterization of MIPs using transgenic mice will be needed to elucidate the functions of individual MIPs. Additionally, genetic analyses of patients with infertility are likely to identify more essential and redundant components.

## STAR METHODS

### RESOURCE AVAILABILITY

#### Lead contact

Further information and requests for resources and reagents should be directed to the lead contact, David A. Agard (david@agard.ucsf.edu).

#### Materials availability

Experimental reagents generated in this study are available from the lead contact with a completed material transfer agreement.

#### Data and code availability

Cryo-EM maps of 48 nm-repeating structures of doublets from wildtype mouse, Tekt5 −/− mouse and human sperm have been deposited in the Electron Microscopy Data Bank (EMDB) with accession codes: EMD-41431, EMD-41320 and EMD-41317, respectively. The EMD-41431 is a composite map with its two submaps deposited with accession codes: EMD-41450 and EMD-41451. Maps of focused refinement of 16 nm-repeating structures of A- and B-tubules from wildtype mouse have been deposited also: EMD-41315 and EMD-41316. The atomic model of the 48-nm repeat of the mouse sperm doublets has been deposited in the Protein Data Bank (PDB) with accession codes 8TO0. MS data are shared and available through the ProteomeXchange Consortium via the PRIDE partner repository under the dataset identifier: PXD036885 (username: reviewer_pxd036885@ebi.ac.uk; password: tMEZ90MC) ^57^. R package source materials for MSstats (version 3) are publicly available through the Krogan Lab GitHub: https://github.com/kroganlab.

After downloading the AlphaFold2 library of the mouse proteome, this code is used to distribute PDB files into subdirectories.


i=0; for f in *; 
do 
## Splitting 50 PDBs in each subdirectory
  d=dir_$(printf %03d $((i/50+1))); 
  mkdir -p $d; 
  mv “$f” $d; 
  let i++; 
done 


This code is used to unbiasedly match all PDBs with the target densities in each subdirectory:


for file in * 
do 
  echo $file 
## CCDC105_flipped_b150.mrc is the target densities, the options could be found in the situs website
  colores ../CCDC105_flipped_b150.mrc ${file} -res 6.0 -cutoff 0.0048 -deg 15.0 
  mkdir ../output/${file}_out 
  mv col_* ../output/${file}_out/. 
  mv 
done 


The cross-correlation scores could then be extracted using the following script:


for f in *.out 
do 
    echo $f 
  grep structure $f/*.pdb >> TheResultFile 
  grep Unnormalized $f/*.pdb >> TheResultFile
done 
grep “correlation” TheResultFile > JustCCResults 


The final output could then be sorted based on the cross-correlation scores in Excel. Note each PDB would be matched to the target densities with multiple orientations, resulting in multiple entries with the same PDB but different cross-correlation scores. The duplicate items for each PDB could be deleted in Excel.

Any additional information required to reanalyze the data reported in this work paper is available from the Lead Contact upon request.

### EXPERIMENTAL MODEL AND STUDY PARTICIPANT DETAILS

#### Mouse models

Wild-type and transgenic male C57BL/6J mice at ages of 10 to 16 weeks were used for imaging in this study. All mice were cared for in compliance with the guidelines outlined in the Guide for the Care and Use of Laboratory Animals. All experiments were approved by the Janelia Research Campus (JRC) IACUC. JRC is an AAALAC-accredited institution. Mice were maintained under SPF conditions.

#### Human sample

A man aged 25–39 years old was recruited and consented to participate in this study. We did not bias ancestry, race or ethnicity throughout the recruitment process. We only checked the samples under the microscope to make sure the sperm were normozoospermic with a cell count of at least 30 million sperm cells per milliliter. All experimental procedures using human-derived samples were approved by the Committee on Human Research at the University of California, Berkeley, under IRB protocol number 2013-06-5395.

### METHOD DETAILS

#### Sample preparation.

Mouse sperm were collected from 10 to 16-week-old C57Bl/6J mice based on the published protocol ^58^. Briefly, the sperm were extracted from vasa deferentia by applying pressure to cauda epididymides in 1x Krebs buffer (1.2 mM KH_2_PO_4_, 120 mM NaCl, 1.2 mM MgSO_4_•7H_2_O, 14 mM dextrose, 1.2 mM CaCl_2_, 5 mM KCl, 25 mM NaHCO_3_). The sperm were washed and resuspended in ~100 μL Krebs buffer for the following experiments. For human sperm samples, freshly ejaculated semen samples were obtained by masturbation.

#### Grid preparation.

EM grids (Quantifoil R 2/2 Au 200 mesh) were glow discharged to be hydrophilic using an easiGlow system (Pelco). The grid was then loaded onto a Leica GP2 plunge freezer (pre-equilibrated to 95% relative humidity at 25 °C). The mouse sperm suspension was then mixed with 10 nm gold beads (Electron Microscopy Science, cat #25487) to achieve final concentrations at 2–6 million cells/mL. EHNA (erythro-9-(2-hydroxy-3-nonyl)adenine) (Santa Cruz Biotechnology, CAS 51350-19-7) was added to a final concentration of 10 mM. Next, 3.5 μL of the sperm mixture was loaded onto each grid, followed by a 15-second incubation period. The grids were then blotted for 4 sec and plunge-frozen in liquid ethane.

#### Cryogenic focused ion beam (cryoFIB) milling.

CryoFIB was performed using an Aquilos II cryo-FIB/SEM microscope (Thermo Fisher Scientific). A panorama SEM map of the whole grid was first taken at 377x magnification using an acceleration voltage of 5 kV with a beam current of 13 pA, and a dwell time of 1 μs. Targets with appropriate thickness for milling were selected on the grid. A platinum layer (~10 nm) was sputter coated and a gas injection system (GIS) was used to deposit the precursor compound trimethyl(methylcyclopentadienyl) platinum (IV). The stage was tilted to 15–20°, corresponding to a milling angle of 8–13° relative to the plane of grids. FIB milling was performed using stepwise decreasing current as the lamellae became thinner (1.0 nA to 30 pA, final thickness: ~300 nm). The grids were then stored in liquid nitrogen before data collection.

#### Image acquisition and tomogram reconstruction.

Tilt series of mouse sperm were collected on a 300-kV Titan Krios transmission electron microscope (Thermo Fisher Scientific) equipped with a high brightness field emission gun (xFEG), a spherical aberration corrector, a Bioquantum energy filter (Gatan), and a K3 Summit detector (Gatan). The images were recorded at a nominal magnification of 26,000x in super-resolution counting mode using SerialEM ^49^. After binning over 2 × 2 pixels, the calibrated pixel size was 2.612 Å on the specimen level. For each tilt series, images were acquired using a modified dose-symmetric scheme between −48° and 48° relative to the lamella with 4° increments and grouping of two images on either side (0°, 4°, 8°, −4°, −8°, 12°, 16°, −12°, −16°, 20°…) ^59^. At each tilt angle, the image was recorded as movies divided into fourteen subframes. The total electron dose applied to a tilt series was 100 e^−^/Å^2^. The defocus target was set to be −2 to −5 μm.

All movie frames were corrected with a gain reference collected in the same EM session. Movement between frames was corrected using MotionCor2 without dose weighting ^60^. All tilt series were aligned using AreTomo and the tomograms were inspected as a screening step to identify good tilt series ^41^. Tilt series with crystalline ice and big ice blocks, or possessing less than five doublets of the axonemes were discarded. Alignment of the good tilt series was then performed in Etomo using the gold beads as fiducial markers ^50^. The AreTomo is less labor-intensive for screening purposes, while the Etomo workflow allowed us to achieve high-resolution reconstructions. The aligned tilt series were then CTF-corrected using TOMOCTF ^51^ and the tomograms were generated using TOMO3D ^52^ (bin4, pixel size: 10.448 Å). In total, we started with eight milling grids of mouse sperm and obtained 159 lamellae. Ultimately, the final reconstructions of the consensus averages were based on 77 usable tomograms.

#### Subvolume averaging.

Subvolume extraction, classification and refinement were first performed using RELION3 as reported previously ^61^. Briefly, subvolumes from the doublets were manually picked every 24 nm and extracted at binning of 6 (pixel size: 15.672 Å, box size: 80 pixels, dimension: 125.376 nm). These subvolumes were aligned to a map of non-treated mouse sperm doublet structure (EMDB-27444) lowpass filtered to 80 Å and the resulting map was used as the reference for further processing. Supervised 3D classification on radial spokes gave rise to four class averages of the 96-nm repeating units at four different registers. All four class averages were recentered at the base of Radial spoke 2 and re-extracted at the same point at the binning of 4 (pixel size: 10.448 Å, box size: 120 pixels, dimension: 125.376 nm). All subvolumes were combined and aligned to one reference and duplicate subvolumes were removed based on minimum distance (< 40 nm). The remaining subvolumes were aligned to yield the consensus average for all nine doublets. Subvolumes of the 96-nm repeating units were recentered on MIP features that repeat every 48 nm or 16 nm to obtain the coordinates of these subvolumes. The tomograms and coordinates were imported in RELION4 without binning ^24^. Pseudo-subtomograms were extracted (pixel size: 2.612 Å, box size: 220 pixels, dimension: 57.464 nm) and the first round of Refine3D jobs yield the initial reference for the following refinement of geometric and optical parameters of the tilt series. TomoFrameAlign and CtfRefineTomo jobs were executed alternatively for two rounds and new pseudo-subtomograms were extracted (see FSC curves in Figure S2A). The same “Refine3D-TomoFrameAlign-CtfRefineTomo-TomoFrameAlign-CtfRefineTomo” process was repeated again and new pseudo-subtomograms were extracted. The final Refine3D job yields the reported maps. Further refinement did not improve the resolutions and quality of maps. In order to generate a map covering the entire 48 nm-repeating structure of the doublets, the run_data.star file from the final Refine3D job was shifted along the longitudinal axis and another round of refinement yielded averages with the shifted register. These reconstructions were aligned to the reported 48 nm repeating structure of doublets from bovine trachea cilia and a composite map was generated to match the register of the periodic structure.

The resolutions of the maps were estimated based on the FSC of two independently refined half datasets (FSC = 0.143). Local resolution maps for doublets of both human and mouse sperm were calculated by RELION4 and displayed in UCSF Chimera ^53^. These local-resolution maps represent relative differences in resolution across the maps but the absolute values may not be precise. IMOD was used to visualize the tomographic slices ^50^. UCSF Chimera was used to manually segment the maps for various structural features and these maps were colored individually to prepare the figures using UCSF ChimeraX ^53,54,62^.

#### Model building and unbiased matching of density maps to protein candidates.

Model building was performed in Coot v0.9.8.1 ^55^ and rigid body fitting was achieved using UCSF Chimera. The interpretation of the mouse sperm doublet map started with the atomic model of the bovine trachea doublet (PDB 7RRO) ^13^. Densities matching tubulins and 29 bovine MIPs in the bovine trachea doublet were found in the mouse sperm doublet map so all of these densities were considered to be formed by *M. musculus* orthologs (Figure S4). We cannot exclude the possibilities that they are formed by sperm-specific homologs with similar tertiary structures. These orthologs were identified using UniProt ^63^ or the NCBI protein database ^64^ based on the sequences of bovine proteins. The atomic models of bovine trachea MIPs were mutated to match the sequence of the mouse proteins using the Chainsaw plugin in Coot. The resulting models of individual proteins were then fit into the mouse sperm doublet map as rigid bodies in Chimera.

MIP densities that are unique in sperm doublets were segmented from the corresponding maps using UCSF Chimera manually. At 6–8 Å resolutions, α-helix is well-resolved and β-strands appear as curved sheets and we focused on the unassigned densities with well-defined tertiary structures or domains (as the various colored densities shown in Figure 1). Meanwhile, the PDB library of 21,615 mouse proteins based on AlphaFold2 prediction was downloaded ^27^. The unbiased matching was carried out using the COLORES program (Situs package, see the code in the Star Methods) ^65^. The matching was scored and ranked by the cross-correlation scores and the top 200 hits were inspected individually with the target densities in UCSF Chimera. We noticed that matching of densities to much larger PDBs could lead to unrealistic cross correlations (>1). Setting the box sizes of the maps to be two or three times larger compared to the isolated densities does not solve the issue since COLORES would cut off the zero valued edges by default to reduce computational loads. We thus used “voledit” command from SITUS to edit the voxels at the corner of the cubic map. Specifically, we edited the first and the last values in the sit file to be slightly larger than the threshold values to avoid the cropping. To test the unbiased proteome-wide search approach, we used densities corresponding to known MIPs identified in bovine tracheal doublets, including PACRG, CFAP20 and NME7 as controls for the method. Indeed, PDB models corresponding to the respective mouse orthologs were identified as the top hits (Data S1), suggesting this visual proteomics approach can identify protein with matching tertiary structures. We then applied this method to identify candidates for the mouse sperm-specific densities (Data S2–4). For the 4-helix bundle densities, the CATH library, which curated non-redundant PDBs of published structural domains ^36^, was also used and no homologous proteins of SPACA9 were found.

The AlphaFold2 predicted PDBs were then used as starting models and initial fitting pose discovered by COLORES were inspected in Chimera. The various Tektin 5 and CCDC105 models were built in Coot to match the corresponding densities. Unresolved loops were deleted. We observed densities corresponding to 24 copies of SPACA9 for the 48 nm-repeating units of the doublet microtubules, albeit with varied occupancies. We rigid-body fitted all 24 SPACA9 in these densities in Coot to reflect the stacking oligomerization in the deposited model. We also performed rigid-body fitting of DUSP3 into the globular densities next to the slanted Tektin 5s. These models were combined in ChimeraX and all side chains were stripped using the *phenix.pdbtools* command.

#### Sequence alignment and search for homologous proteins.

Sequence alignment was performed using Clustal Omega ^32^ server and displayed in Jalview ^66^. *M. musculus* Tektin 1 sequence was used as input to search for Tektin homologs using the HHpred server ^67^.

#### Biochemical extractions of mouse sperm.

For each of the three biological replicates, sperm from two mice were washed with PBS and pelleted at 2000x g for 5 min. Then, the E1-E5 buffers were used to extract proteins from the pellets [0.1 % Triton in PBS (E1), 0.6 M NaCl in PBS (E2), 0.6 M KCSN in PBS (E3), 8 M urea (E4) and 10% SDS (E5)]. For E1 to E4, 100 μL of the buffer was added to the pellets and the resuspension was mixed by pipetting up and down using a p200 pipette. Then the solution was incubated at room temperature for 10 min and the pellet was spun down at 21,000x g for 10 min. For E5, after 10% SDS was added and mixed, the resuspension was heated at 95° for 5 min. After the pellets were spun down, the supernatant was taken as the extraction. 20 μL and 2 μL of the extractions were used for SDS-PAGE analyses, either stained with AcquaStain (Fisher Scientific, NCO170988) and blotted with an antibody against α-tubulins (ThermoFisher Scientific, DM1A, #62204). The remaining extractions were used for mass spectrometry analysis.

#### Mass spectrometry (MS)-based global protein abundance of mouse sperm.

Proteins in biochemical fractions E1, E2, E3, E4 and E5 from three biological replicates were reduced and alkylated in 4 mM final concentration tris (2-carboxyethyl) phosphine (TCEP) and 10 mM final concentration iodoacetamide by 20-minute incubation in the dark, after which excess iodoacetamide was quenched with 10 mM final concentration dithiothreitol (DTT). Proteins were then subjected to methanol chloroform precipitation. Briefly, 1 part sample was combined and vortexed sequentially with 4 parts methanol, 1 part chloroform, and 3 parts water for phase separation, after which samples were spun for 2 minutes at top speed (14,000 g) in a bench-top centrifuge (Centrifuge 5424R, Eppendorf). The upper phase was removed and discarded, and 4 parts methanol were combined and vortexed with the interphase and lower phase and subsequently centrifuged for 3 minutes at 14,000 g. The supernatant was removed and discarded, and the pellet was washed three times in 80% ice-cold acetone followed by centrifugation for 3 minutes at 14,000 g. Extracted proteins were air dried, resuspended in 8 M urea buffer (8 M urea, 150 mM NaCl, 50 mM NH_4_HCO_3_, cOmplete Mini EDTA-free protease inhibitor (Roche, 11836170001)), and quantified using Bradford reagent (Sigma, B6916) following Coomassie (Bradford) Protein Assay Kit’s protocol (Thermo Fisher, 23200). Following quantification, protein samples were diluted 4-fold to 2 M urea concentration with 0.1 M NH_4_HCO_3_ pH 8, digested with trypsin (Promega, V5111) at a protease:protein ratio of 1:100 (weight/weight), and incubated overnight at 37°C in a thermomixer at 750 rpm.

After tryptic digest, samples were acidified to pH <3 with 1% final concentration formic acid, and desalted for MS analysis using HPLC-grade reagents and 100 μL OMIX C18 tips (Agilent Technologies, A57003100) according to the manufacturer’s protocol with the following adjustments. Briefly, OMIX tips were conditioned by sequential washes of 100% acetonitrile and 50% acetonitrile, 0.1% formic acid, and equilibrated with two washes of 0.1% formic acid. Peptides were bound to the C18 polymer by repeated pipetting, subsequently washed three times with 0.1% formic acid, and sequentially eluted in 50% acetonitrile, 0.1% formic acid followed by 90% acetonitrile, 0.1% formic acid. Peptides were dried by vacuum centrifugation (CentriVap Cold Trap, Labconco) and stored at −80°C until MS analysis.

Digested, desalted peptides were resuspended to 0.125–2 μg/μL final concentration in 2% acetonitrile, 0.1% formic acid. 1–2 μL were injected in technical singlet onto an Easy-nLC 1200 (Thermo Fisher Scientific) interfaced via a nanoelectrospray source (Nanospray Flex) coupled to an Orbitrap Fusion Lumos Tribrid mass spectrometer (Thermo Fisher Scientific). Peptides were separated on a PepSep reverse-phase C18 column (1.9 μm particles, 1.5 μm × 15 cm, 150 μm ID) (Bruker) with a gradient of 5–88% buffer B (0.1% formic acid in acetonitrile) over buffer A (0.1% formic acid in water) over a 100-minute data acquisition. Spectra were acquired continuously in a data-dependent manner. One full scan in the Orbitrap (scan range 350–1350 m/z at 120,000 resolution in profile mode with a custom AGC target and maximum injection time of 50 milliseconds) was followed by as many MS/MS scans as could be acquired on the most abundant ions in 2 seconds in the dual linear ion trap (rapid scan type with fixed HCD collision energy of 32%, custom AGC target, maximum injection time of 50 milliseconds, and isolation window of 0.7 m/z). Singly and unassigned charge states were rejected. Dynamic exclusion was enabled with a repeat count of 1, an exclusion duration of 25 seconds, and an exclusion mass width of ±10 ppm. Liquid chromatography ^68^ and MS acquisition parameters are reported in (table S1).

Raw MS files were searched using MaxQuant (version 1.6.3.3) against a database of the mouse proteome (SwissProt Mus musculus reviewed protein sequences, downloaded 07 May 2022) with a manual addition to include mouse piercer of microtubule wall 2 protein (protein sequence from NCBI Reference Sequence NP_001185718.1, manually assigned the UniProt identifier “ZCC15orf65” in our database after its bovine homolog) ^37^. MaxQuant settings were left at default, with the following exceptions: LFQ was enabled with skip normalization enabled; and match between runs was enabled with a 1.5-minute matching time window and 20-minute alignment window. Trypsin (KR|P) was selected and allowed up to two missed cleavages, and variable and fixed modifications were assigned for protein acetylation (N-terminal), methionine oxidation and carbamidomethylation.

Statistical analysis of protein quantitation was completed with R Bioconductor package artMS (version 1.14.0) ^56^ and its function artmsQuantification, which is a wrapper around the R Bioconductor package Mass Spectrometry Statistics and Quantification (MSstats) (version 4.4.0) as follows ^38^ (table S2). Peptide intensities from the MaxQuant evidence file were summarized to protein intensities using the MSstats function dataProcess with default settings. The differences in log2-transformed intensity between biochemical fractions were scored using the MSstats function groupComparison, which fits a single linear model for each protein with a single categorical variable for condition, or fraction in our case. From these models, MSstats reports pairwise differences in means between conditions as log2 fold change (log2FC) with a p-value based on a t-test assuming equal variance across all conditions, and reports adjusted p-values using the false discovery rate (FDR) estimated by the Benjamini-Hochberg procedure. Proteins with significant changes in abundance between fractions were defined as: (1) absolute(log2FC) > 1; and (2) adjusted p-value < 0.05. Proteins with significant changes in abundance were tested for enrichment of Gene Ontology terms (table S3). The over-representation analysis was performed using the enricher function from R package clusterProfiler (version 4.4.1) ^39^. Gene Ontology (GO Biological Process, Molecular Function and Cellular Component) terms and annotations were obtained from the R annotation package org.Mm.eg.db (version 3.15.0). From among all significantly enriched terms, we selected a set of non-redundant terms following a clustering procedure. We first constructed a term tree based on distances (1-Jaccard Similarity Coefficients of shared genes in KEGG or GO) between the significant terms. The term tree was cut at a specific level (h = 0.99) to identify clusters of non-redundant gene sets (table S4). For results with multiple significant terms belonging to the same cluster, we selected the most significant (lowest adjusted p-value) term.

#### Generation of Tektin-5 knockout mice and functional/structural analyses of mutant sperm.

To create an easily detected frameshift mutation in the *Tekt5* gene, we used two gRNAs located in exon 1 of the gene. There is a pseudogene located on chromosome 19 with 85.8% homology to the Tektin5 coding region. Three gRNAs were carefully selected to avoid cutting the pseudogene. They are gRNA1 (CGCTGGGTCTCCACGCGTTCAGG), gRNA2 (AGTTTCTGTGGCCCCAAGAAAGG), and gRNA3 (CCGAGGAATGCTCAGGCATCCGG). The gRNAs were in vitro transcribed using the MEGA shortscript T7 kit (Life Tech Corp AM1354).

Two combinations of the gRNAs were used: gRNA1 + gRNA2, and gRNA2 + gRNA3. The gRNAs and Cas9 protein (Invitrogen Truecut Cas9 protein V2, cat#A36498) with a concentration of 125 ng each were co-electroporated into 1-cell C57Bl/6J embryos using a BEX Genome Editor. A total of 75 pups were weaned and genotyped by PCR. Thirty-nine of them with an obviously shorter PCR band were sequenced, and nine of them with a frameshift deletion were selected for germline testing. Germline transmission was found in 7 out of 9, and 2 lines were selected for further breeding: line 6 with a 166 bp deletion and line 7 with a 181 bp deletion. Both lines were then mated with wild-type females to generate F1 animals. Mating of heterozygous F1 male and female led to F2 homozygotes, which were further confirmed by sequencing.

Sperm motility was recorded at 37°C on a Hamilton Thorne IVOS II CASA machine using a Zeiss 10x NH objective, at a frame rate of 60 Hz, in the presence of 1% polyvinyl alcohol to prevent cell adhesion to glass. The motility and sperm morphologies were inspected and counted manually.

The structural analyses of the mutants were performed using the same workflow described for wild-type sperm, except omitting the EHNA treatment. Sperm from two different Tektin5-knockout lines were processed, imaged and analyzed independently. The two independent reconstructions show consistently low occupancies of specific Tektin 5 densities and only the higher-resolution reconstruction is shown.

### QUANTIFICATION AND STATISTICAL ANALYSIS

Statistical analyses of *Tekt5* −/− mouse were prepared using Prism9 (GraphPad). We performed six mating trials by using six *Tekt5* −/− males (3 from each line of two lines) and six wildtype females. The average and standard deviation are presented (7.3 ± 1.4, N=6). For the sperm analyses, we counted enough videos so the number of sperm from each mouse is > 200. Sperm from three *Tekt5* −/− knockout mutants and two wild-type mice were analyzed functionally.

## Supplementary Material

1 **Data S1. Unbiased matching of densities corresponding to known MIPs in mouse sperm doublet with a library of mouse proteome with 21615 PDBs predicted by AlphaFold2. Related to Figure 2 and STAR Methods**. Densities corresponding to PACRG (**A**), CFAP20 (**B**), and NME7 (**C**) were used as positive controls for the unbiased matching workflow. The top 10 hits based on cross-correlation scores (CC) from COLORES are ranked. The COLORES outputs multiple possible different orientations for each match but only the best poses are shown with the target densities. The corresponding PDBs were all found to be the best hits. Although the individual β-strands in CFAP20 are not resolved, the shapes of the β-sheets are clearly distinct from α-helices and could be matched with the correct PDB models. For the densities corresponding to PACRG, the 2^nd^ hit is PACRL (PACRG-like protein), which was not found in our mass spectrometry analyses of mouse sperm.

2 **Data S2. Unbiased matching of 3-helix densities at the ribbon of the mouse sperm doublet with a library of mouse proteome with 21615 PDBs predicted by AlphaFold2. Related to Figure 2. Related to Figure 2 and STAR Methods.** The top 30 hits based on cross-correlation scores (CC) from COLORES are ranked. The COLORES outputs multiple possible different orientations for each match but only the best poses are shown with the target densities. CCDC105 and Tektin 1–5 matches the secondary structures of the target densities. However, the other proteins match the overall shapes but not the features of secondary structures at 6–7 Å resolutions. Upon manual inspections, CCDC105 matches the lengths and orientations of the helices better than Tektin 1–5. Also, the well-defined densities corresponding to the conserved proline-rich loop in CCDC105 is distinct from densities of Tektins. Note the orientations of the Tektin 1–5 and CCDC105 are not the same and other poses of these proteins were also considered when building the models.

3 **Data S3. Unbiased matching of 4-helix densities in the B-tubule of the mouse sperm doublet with a library of mouse proteome with 21615 PDBs predicted by AlphaFold2. Related to Figure 2 and STAR Methods**. The top 30 hits based on cross-correlation scores (CC) from COLORES are ranked. The COLORES generates multiple possible different orientations for each match but only the best poses are shown with the target densities. SPACA9 matches the secondary structures of the target densities, while most of the other proteins match the overall shapes but not the features of secondary structures at 6–7 Å resolutions. The 15^th^ hit, SGMR2, only partially matches for the secondary structure and is a transmembrane protein.

4 **Data S4. Unbiased matching of bent helical densities in the A-tubule of the mouse sperm doublet with a library of mouse proteome with 21615 PDBs predicted by AlphaFold2. Related to Figure 2 and STAR Methods**. The top 30 hits based on cross-correlation scores (CC) from COLORES are ranked. The COLORES outputs multiple possible different orientations for each match but only the best poses are shown with the target densities. Tektin 1–5 and CCDC105 match most of the secondary structures of the target densities, apart from the missing single helix. The other proteins match the overall shapes but not the features of secondary structures at 6–7 Å resolutions.

5

6

7

8

9 **Figure S1. Workflow of data processing, related to Figures 1, 2, and 3 and STAR Methods**. (A) Tilt series comprised of 25 2D projections were recorded. The image shows the midpiece of sperm flagella that contains mitochondria around the axoneme. Gold beads on the tilt images are indicated (green arrowhead). The alignment of gold beads was used to align the tilt images. (B) 3D tomograms were reconstructed and subvolumes were picked along the microtubules. (C) 3D classification and refinement were performed to align and average the subtomogram for the 96 nm-repeating structures of the mouse sperm doublets. Four views of the 96 nm-repeating structure of doublets from EHNA-treated sperm are shown for the 3D reconstruction generated using RELION3 as reported previously ^19^. Gold-standard Fourier Shell Correlation (FSC) curve calculated between half maps of mouse sperm doublets. The resolution was estimated as 26 Å (FSC = 0.143). (D) Two slices of the 96 nm-repeating structure of doublets looking along and perpendicular to the filament axis. Note the red line in the top panel indicates the plane of the bottom slice and periodic structures are observed inside the microtubules. The coordinates were recentered on the 48-nm repeats and imported into RELION4. In the top panel, note the features further away from the microtubules are blurrier, suggesting that there are conformational heterogeneities and they are resolved at lower resolutions. (E) The initial Refine3D job of the 48-nm repeating structures was performed using RELION4 ^24^. (F) The 3D reconstructions were matched to the 2D projections of individual particles in the raw tilt images and this step refined both the geometric and optical parameters of the tilt series. (G) Another round of subtomogram averaging was performed based on refined tilt series. No additional improvement was observed after 3 rounds of refinement and Refine3D as shown in (F)-(G).

10 **Figure S2. Characterization of the 48 nm-repeating structure of doublets from mouse sperm. Related to Figures 1 and 3**. (**A**) Gold-standard Fourier Shell Correlation (FSC) curves were calculated between half maps of mouse sperm doublets. The resolutions were reported as FSC = 0.143. Note the FSC curves resulting from the iterative frame alignment and CTF refinement between the second and third Refine3D jobs were not shown for the clarity of the figure. Further refinement after the third Refine3D did not improve the resolution or quality of the map. (**B**) The local-resolution map of mouse sperm doublets was calculated by RELION4. The ribbon region has the highest resolutions. Densities in the A-tubule have higher resolutions than the ones from the B-tubule. (**C**) Equivalent longitudinal cross-section views of doublets from mouse sperm and bovine trachea cilia (EMD-24664) are shown ^13^. The latter was low-pass filtered to 7.5 Å and comparable details of the secondary and tertiary structures of the MIPs are observed. (**D**) The reconstruction of mouse sperm doublet (grey) is overlaid with the bovine trachea doublets (yellow). The mouse sperm-specific densities are highlighted (dashed ovals). The broken helical bundles and the curved helical bundles inside the A-tubule of mouse sperm doublets along the microtubule axis are shown. The discontinuous parts of the broken helical bundles are indicated (dashed rectangles). Note the curved bundles have one straight and two curved groups of densities in every 48-nm repeat (outlined using dashed shapes).

11 **Figure S3. Characterization of the 48 nm-repeating structure of doublets from human sperm. Related to Figure 1**. (**A**) A gold-standard Fourier Shell Correlation (FSC) curve was calculated between half maps of mouse sperm doublets. The resolution was estimated as 10.3 Å (FSC = 0.143). (**B**) The local-resolution map of human sperm doublets was calculated by RELION4. The ribbon region has the highest resolutions. Densities in the A-tubule have higher resolutions than the ones from the B-tubule. (**C**) Equivalent views of doublets from human sperm and bovine trachea cilia (EMD-24664) are shown ^13^. The latter was low-pass filtered to 10 Å and comparable details of the secondary and tertiary structures of the MIPs are observed. (**D**) The reconstruction of human sperm doublet (blue) is overlaid with the bovine trachea doublets (yellow) at low and high thresholds. (**E**) The two broken bundles inside the A-tubule in human sperm are shown at a low threshold (see the corresponding mouse densities in Figure S2D). (**F**) The curved helical bundles contain one straight and two curved groups of densities inside the A-tubule of human sperm are outlined. Human sperm-specific densities were observed to connect one curved bundle to the lumen of A-tubule. (**G**) The human sperm doublets overlaid with mouse sperm doublets are shown. The inconsistent densities are outlined (dashed line) (also see Figures S2D and S3F).

12 **Figure S4. Rigid-body fitting of 29 identified MIPs from bovine trachea cilia into the density map of mouse sperm doublet. Related to Figures 2, 3 and STAR Methods**. (**A**)-(**F**), Models of 29 known MIPs from bovine trachea cilia (PDB 7RRO) ^13^ are fitted into the density map of mouse sperm doublet. The viewing angles for all panels are shown. For proteins that have multiple α-helices (CFAP161, RIBC2, CFAP53, MNS1, CFAP21, NME7, CFAP141, EFHC1, EFHC2, ENKUR, CFAP210, EFCAB6, CFAP45, PACRG and TEKTIN 1–4), the arrangement of secondary structures matches densities in sperm doublets. The overall shapes of β-sheet-rich proteins (CFAP52 and CFAP20) match the densities and these proteins are highly conserved in axonemes. For the proteins that contain random coils, we did observe matching features in the maps but it is generally harder to trace the main chains at the current resolution (CFAP95, SPAG8, CFAP107, FAM166B, Pierce1, Pierce2, CFAP126, CFAP276 and TEKTIP1).

13 **Figure S5. Characterization of the 16 nm-repeating structures of doublets from mouse sperm. Related to Figure 2**. (**A**)-(**B**) Gold-standard Fourier Shell Correlation (FSC) curves were calculated using half maps of 16 nm-repeating structures of A-tubule and B-tubule. The resolution was estimated as 6.0 Å and 6.7 Å, respectively (FSC = 0.143). The Nyquist limit is 5.30 Å. (**C**)-(**E**), The local resolution map was calculated from the two half maps of 16 nm-repeating structures of A-tubule using RELION4. The viewing angles for (**D**) and (**E**) are shown in (**C**) (black arrow). These viewing angles are similar to Figures 1A, 1D and 1E, respectively. (**F**)-(**H**), The local resolution map was calculated using half maps of 16 nm-repeating structures of B-tubule using RELION4. The viewing angles for (**G**) and (**H**) are shown in (**F**) (black arrow). The viewing angles of (**F**) and (**G**) are similar to Figure 1A and F, respectively.

14 **Figure S6. Tektin 5 and CCDC105 likely form sperm-specific 3-helix bundles associated with the A-tubule. Related to Figures 2 and 3**. (**A**) After unbiased matching, Tektin 5 was scored as the #5 hit of the predicted structures out of 21,615 proteins from the mouse proteome, ranked by cross-correlation scores (Top 10 are shown). Tektin 1–4 were ranked at #7–10 due to their similar tertiary structures. (**B**) Typical false positives (#1–4 and #6) from the same search. Usually, these are proteins with long single helices that do not match the gaps observed in the map. Also, they do not explain the 3-helix bundles. The fitting of Tektin 5 into the same densities is shown for comparison. (**C**) The structure of CCDC105 directly predicted by AlphaFold2 (left) is compared to the predicted complex formed by two CCDC105 molecules (right). The full-length CCDC105 molecule in the complex is colored based on the per-residue confidence scores (predicted local distance difference test, or pLDDT) from the AlphaFold2 prediction. The three P-loops have medium confidence scores (green), suggesting the exact conformations of these loops may not be accurately predicted. However, the presence of these structured loops is conceivably confident based on the conserved proline residues (see the sequence alignment in (**D**)) and matched the protrusion densities observed in our maps (Figure 2G). Note the conformations of the three proline-rich loops differ in these two predictions. These differences could be caused by the presence of neighboring molecules ^27^. (**D**) The sequence alignment of CCDC105 from five mammals (*H. sapiens*, *M. musculus*, *B. taurus*, *S. scrofa* and *F. catus*), zebrafish (*D. rerio*) and sea urchins (*S. purpuratus*). The three proline-rich loops are marked above the sequences. (**E**) The models of CCDC105 and Tektin 5 are fitted into the densities of the 3-helix bundle at the ribbon, where the former model explains the extra protrusions and orientation/lengths of helices of the densities but the latter does not.

15 **Figure S7. DUSP proteins in the A-tubule. Related to Figure 3**. (**A**) At a lower threshold compared to Figure 3C, densities connecting the N-terminal residues of the slanted Tektin 5s (magenta models) and the DUSPs (blue models) are observed. (**B**) The DUSP3 is fitted into the globular domain and three orthogonal views are shown. Other homologous DUSP proteins fit well into the density because of similar tertiary structures (DUSP 3, 13, 14, 18, 21 and 29).

16 **Figure S8. Biochemical extractions of proteins from mouse sperm. Related to Figure 3**. (**A**) SDS-PAGE analyses of protein extractions from mouse sperm using 0.1 % Triton in PBS (E1), 0.6 M NaCl in PBS (E2), 0.6 M KCSN in PBS (E3), 8 M urea (E4) and 10% SDS (E5). (**B**) Western blot analyses of protein extractions from mouse sperm using an antibody against α-tubulins. Note strong bands were detected only in E3 and E4, suggesting the microtubule structures were stable in Triton and high NaCl buffer, and dissembled completely in KCSN/urea solutions. (**C**) Bar chart of the number of proteins identified by MS (Protein Count) in each fraction (E1-E5) and biological replicate. We identified a total of 1,677 mouse proteins, with a range of 772 to 1,326 proteins identified in each individual fraction and replicate. (**D**) Heatmap of proteins with significant changes between any two fractions (absolute log2FC > 1, adjusted p-value < 0.05), listed by fractions (E1-E5) and biological replicate and clustered by correlation of intensity profile. Proteins are colored by the log2 fold change (log2FC) in protein intensity normalized to the row median (red, increased intensity; blue, decreased intensity; grey, not detected). Cluster identification numbers (Cluster ID) are labeled (left). (**E**) Heatmap of gene ontology (GO) enrichments among the significantly changing proteins identified in each cluster from (**D**) (left to right: Cluster ID 1–6, as labeled in **D**). GO terms were curated from the top 4 enrichment terms per cluster, and non-redundant terms were selected by an automated clustering procedure (see Materials and Methods). Increased shading reflects increased significance of the enrichment term. The number of proteins per enrichment term is shown in white if significant (adjusted p-value < 0.05), and grey if not significant (adjusted p-value > 0.05). A bar chart plotting the number of total genes in each cluster ID is included ^48^. (**F**), Log2 protein intensities (y-axis) for eight mouse proteins as quantified by MS in each fraction (E1-E5) and biological replicate (colored dots; maximum n=3).

## Figures and Tables

**KEY RESOURCES TABLE:** 

REAGENT or RESOURCE	SOURCE	IDENTIFIER
Biological samples		
Mouse (*Mus musculus*) sperm	Gene Targeting and Transgenics Center, Janelia Research Campus	N/A
Human (*Homo sapiens*) sperm	Lishko Laboratory, UC Berkeley	N/A
Chemicals, peptides, and recombinant proteins		
NaCl	Sigma-Aldrich	Cat #71376
KH_2_PO_4_	Sigma-Aldrich	Cat #P0662
MgSO_4_•7H_2_O	Sigma-Aldrich	Cat #M3409
Dextrose	Sigma-Aldrich	Cat # D9434
CaCl_2_	Sigma-Aldrich	Cat #21097
KCl	Sigma-Aldrich	Cat #60128
NaHCO_3_	Sigma-Aldrich	Cat #S5761
DTT	Sigma-Aldrich	Cat #DTT-RO
TCEP	Sigma-Aldrich	Cat #C4706
erythro-9-(2-hydroxy-3-nonyl)adenine	Santa Cruz Biotechnology	Cat #sc-201184
KCSN	Sigma-Aldrich	Cat #60178
Urea	Sigma-Aldrich	Cat #U5128
SDS	Sigma-Aldrich	Cat #62862
Triton	Sigma-Aldrich	Cat #X100PC
Deposited data		
Cryo-EM map of the 16 nm-repeating A-tubule (mouse)	This paper	EMD-41315
Cryo-EM map of the 16 nm-repeating B-tubule (mouse)	This paper	EMD-41316
Cryo-EM map of the 48 nm-repeating doublets (mouse), the composite map	This paper	EMD-41431
Cryo-EM map of the 48 nm-repeating doublets (mouse), submap 1 of EMD-41431	This paper	EMD-41450
Cryo-EM map of the 48 nm-repeating doublets (mouse), submap 2 of EMD-41431	This paper	EMD-41451
Cryo-EM map of the 48 nm-repeating doublets (human)	This paper	EMD-41317
Cryo-EM map of the 48 nm-repeating doublets (*Tekt5* −/−)	This paper	EMD-41320
Model of the mouse sperm doublets	This paper	PDB: 8TO0
PRIDE partner repository for MS data	This paper	PXD036885
R package source materials for MSstats from Krogan Lab	This paper	https://github.com/kroganlab
Experimental models: Organisms/strains		
Mouse: C57BL/6J	The Jackson Laboratory	https://www.jax.org/strain/000664
*Tekt5* −/− mouse	This paper	N/A
Software and algorithms		
Prism v8	GraphPad	https://www.graphpad.com/
SerialEM 3.8	Mastronarde^49^	https://bio3d.colorado.edu/SerialEM/
Etomo	Kremer et al.^50^	https://bio3d.colorado.edu/imod/doc/UsingEtomo.html
TOMOCTF	Fernandez et al.^51^	https://sites.google.com/site/3demimageprocessing/tomoctf
TOMO3D 2.0	Agulleiro and Fernandez^52^	https://sites.google.com/site/3demimageprocessing/tomo3d
Chimera	Pettersen et al.^53^	https://www.cgl.ucsf.edu/chimera/
ChimeraX	Goddard et al.^54^	https://www.rbvi.ucsf.edu/chimerax/
RELION-4.0	Zivanov et al.^24^	https://relion.readthedocs.io/en/release-4.0/
AlphaFold2	Jumper et al.^27^	https://alphafold.ebi.ac.uk/
Situs	Wriggers et al.^28^	https://situs.biomachina.org/fguide.html
Coot 0.9.8.1	Emsley et al.^55^	http://www2.mrc-lmb.cam.ac.uk/personal/%20pemsley/coot
MaxQuant 1.6.3.3	Cox and Mann^37^	https://www.maxquant.org/
R Bioconductor package artMS 1.14.0	Jimenez-Morales, D. et al.^56^	doi:10.18129/B9.bioc.artMS
Other		
Quantifoil holey carbon grids (R2/2, 200-mesh gold)	Quantifoil MicroTools GmbH	https://www.quantifoil.com/products
EM GP2 Automatic Plunge Freezer	Leica Microsystems	https://www.leica-microsystems.com/products

## HIGHLIGHTS

*In situ* cryoET revealed native structures of doublet microtubules in mammalian sperm

Subtomogram averaging led to 6-Å reconstruction of microtubule doublets

Protein discovery by matching structures to the AlphaFold2-predicted mouse proteome

Sperm doublets feature a plastic and partially redundant Tektin 5 network

## INCLUSION AND DIVERSITY

We support inclusive, diverse, and equitable conduct of research.
