# Supplementary figures and images for: De novo protein identification in mammalian sperm using in situ cryo-electron tomography and AlphaFold2 docking
Source: Cell. Author manuscript; Available in PMC 2024 Feb 5. (PMC10842264; doi:10.1016/j.cell.2023.09.017)

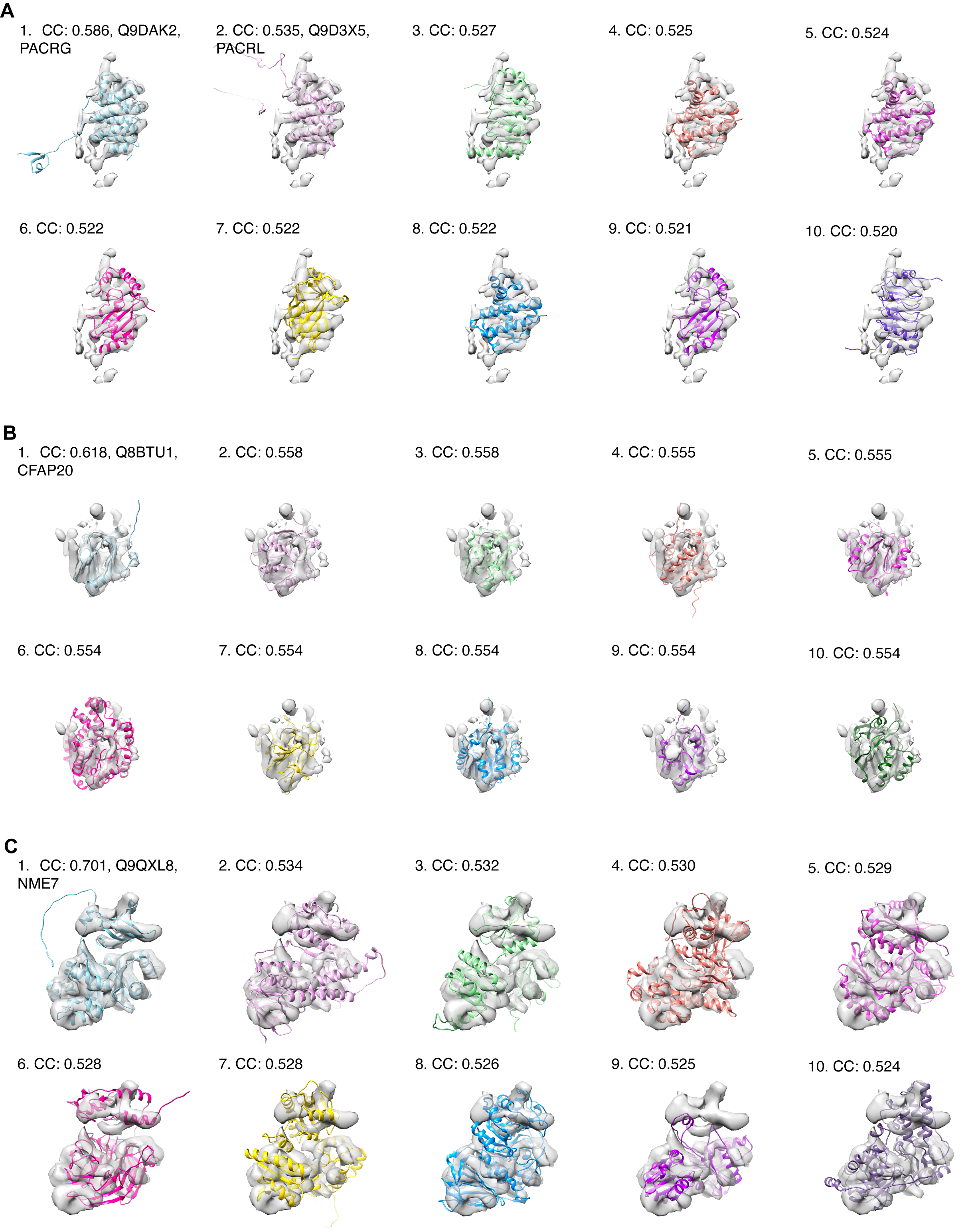

Supplement: 1 — Data S1. Unbiased matching of densities corresponding to known MIPs in mouse sperm doublet with a library of mouse proteome with 21615 PDBs predicted by AlphaFold2. Related to Figure 2 and STAR Methods. Densities corresponding to PACRG (A), CFAP20 (B), and NME7 (C) were used as positive controls for the unbiased matching workflow. The top 10 hits based on cross-correlation scores (CC) from COLORES are ranked. The COLORES outputs multiple possible different orientations for each match but only the best poses are shown with the target densities. The corresponding PDBs were all found to be the best hits. Although the individual β-strands in CFAP20 are not resolved, the shapes of the β-sheets are clearly distinct from α-helices and could be matched with the correct PDB models. For the densities corresponding to PACRG, the 2nd hit is PACRL (PACRG-like protein), which was not found in our mass spectrometry analyses of mouse sperm. [file NIHMS1939567-supplement-1.tif]

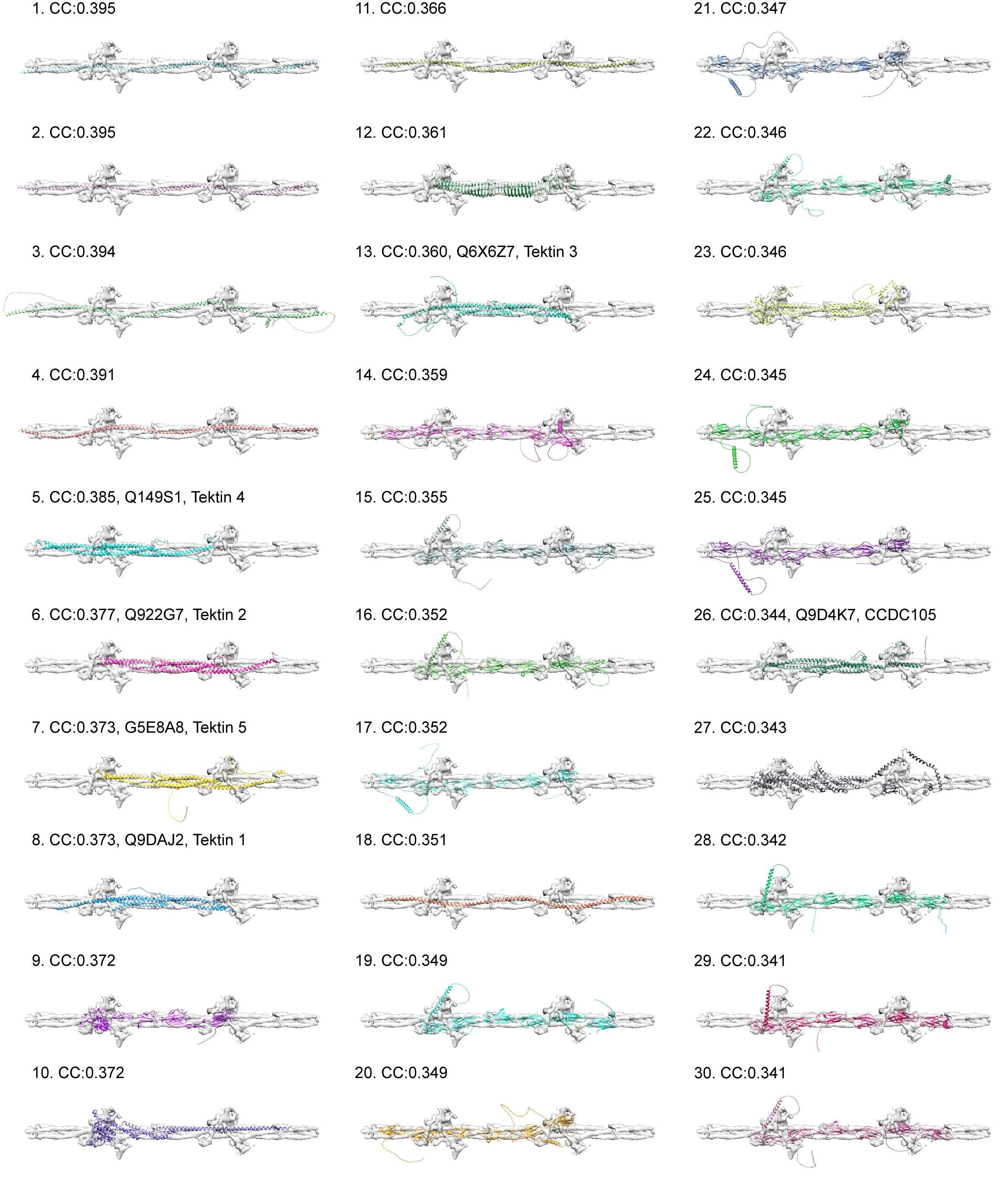

Supplement: 2 — Data S2. Unbiased matching of 3-helix densities at the ribbon of the mouse sperm doublet with a library of mouse proteome with 21615 PDBs predicted by AlphaFold2. Related to Figure 2. Related to Figure 2 and STAR Methods. The top 30 hits based on cross-correlation scores (CC) from COLORES are ranked. The COLORES outputs multiple possible different orientations for each match but only the best poses are shown with the target densities. CCDC105 and Tektin 1–5 matches the secondary structures of the target densities. However, the other proteins match the overall shapes but not the features of secondary structures at 6–7 Å resolutions. Upon manual inspections, CCDC105 matches the lengths and orientations of the helices better than Tektin 1–5. Also, the well-defined densities corresponding to the conserved proline-rich loop in CCDC105 is distinct from densities of Tektins. Note the orientations of the Tektin 1–5 and CCDC105 are not the same and other poses of these proteins were also considered when building the models. [file NIHMS1939567-supplement-2.tif]

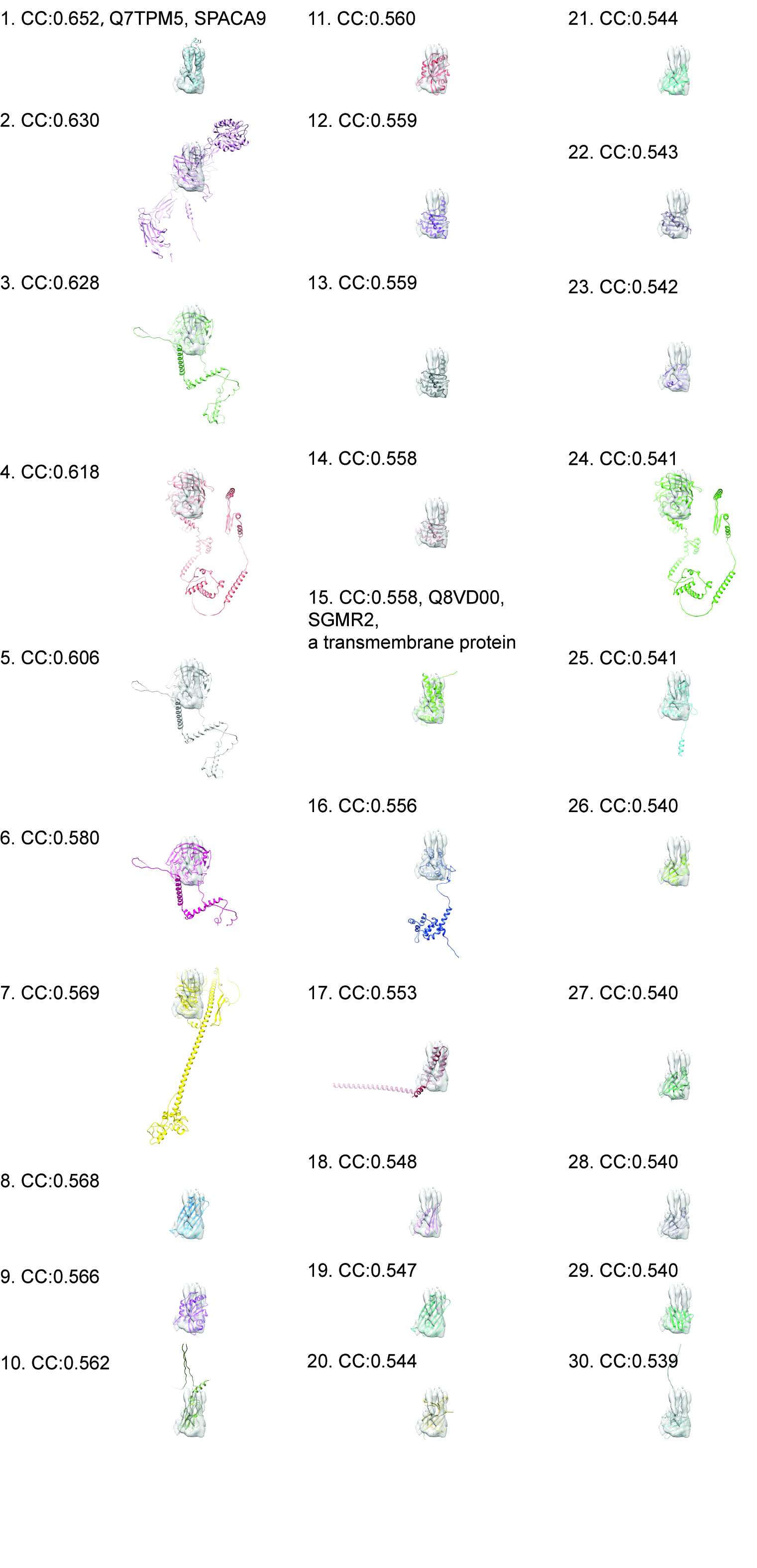

Supplement: 3 — Data S3. Unbiased matching of 4-helix densities in the B-tubule of the mouse sperm doublet with a library of mouse proteome with 21615 PDBs predicted by AlphaFold2. Related to Figure 2 and STAR Methods. The top 30 hits based on cross-correlation scores (CC) from COLORES are ranked. The COLORES generates multiple possible different orientations for each match but only the best poses are shown with the target densities. SPACA9 matches the secondary structures of the target densities, while most of the other proteins match the overall shapes but not the features of secondary structures at 6–7 Å resolutions. The 15th hit, SGMR2, only partially matches for the secondary structure and is a transmembrane protein. [file NIHMS1939567-supplement-3.tif]

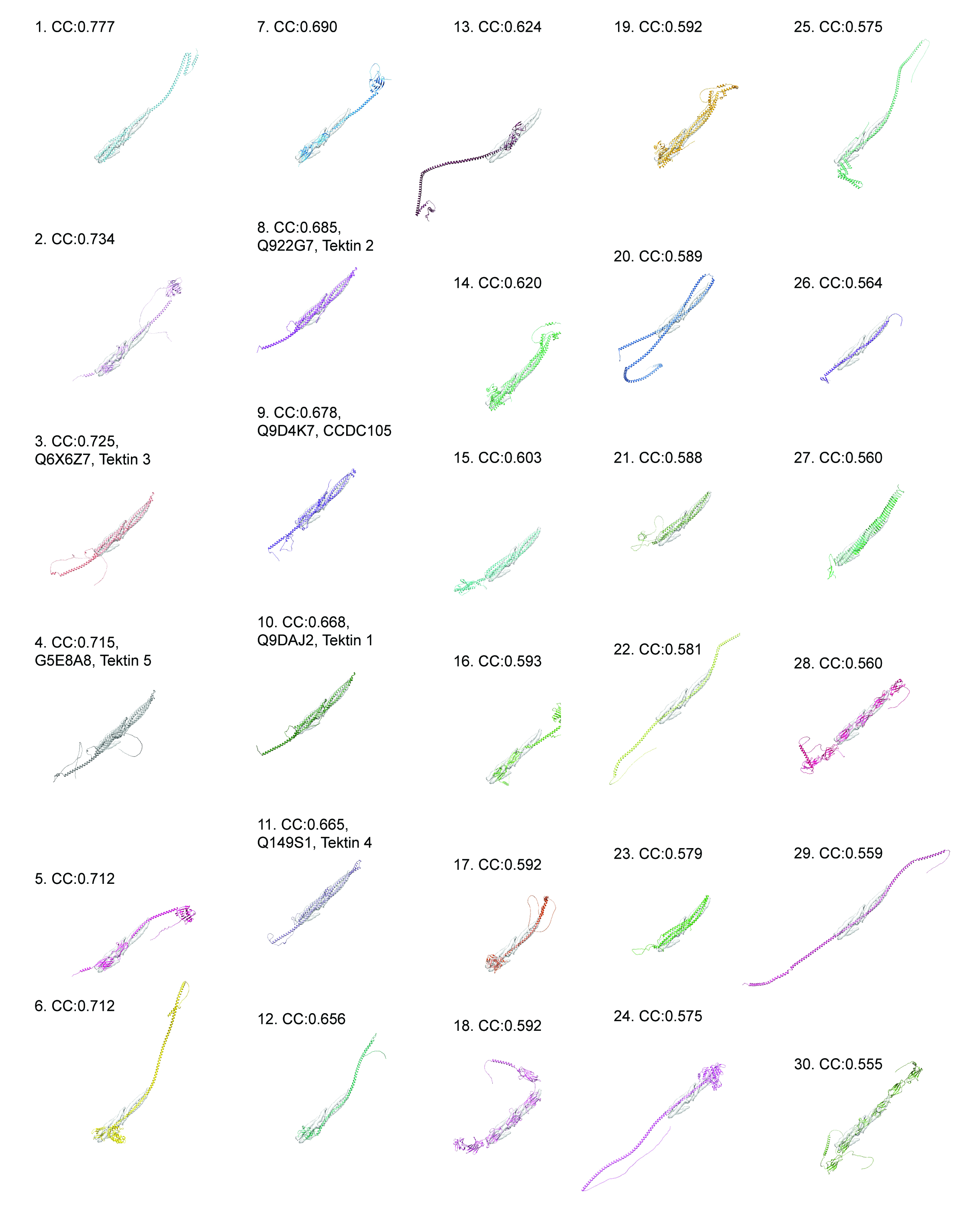

Supplement: 4 — Data S4. Unbiased matching of bent helical densities in the A-tubule of the mouse sperm doublet with a library of mouse proteome with 21615 PDBs predicted by AlphaFold2. Related to Figure 2 and STAR Methods. The top 30 hits based on cross-correlation scores (CC) from COLORES are ranked. The COLORES outputs multiple possible different orientations for each match but only the best poses are shown with the target densities. Tektin 1–5 and CCDC105 match most of the secondary structures of the target densities, apart from the missing single helix. The other proteins match the overall shapes but not the features of secondary structures at 6–7 Å resolutions. [file NIHMS1939567-supplement-4.tif]

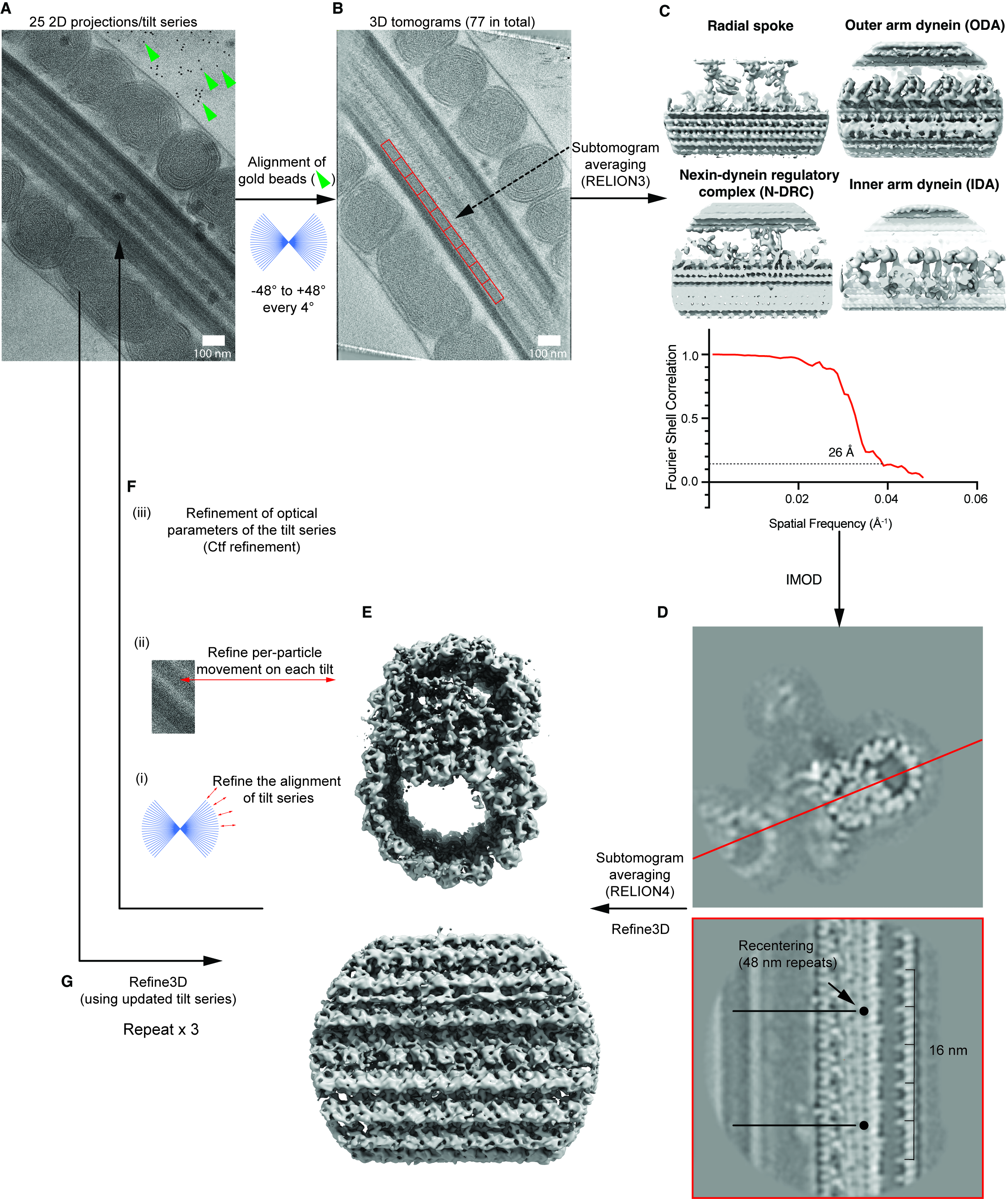

Supplement: 9 — Figure S1. Workflow of data processing, related to Figures 1, 2, and 3 and STAR Methods. (A) Tilt series comprised of 25 2D projections were recorded. The image shows the midpiece of sperm flagella that contains mitochondria around the axoneme. Gold beads on the tilt images are indicated (green arrowhead). The alignment of gold beads was used to align the tilt images. (B) 3D tomograms were reconstructed and subvolumes were picked along the microtubules. (C) 3D classification and refinement were performed to align and average the subtomogram for the 96 nm-repeating structures of the mouse sperm doublets. Four views of the 96 nm-repeating structure of doublets from EHNA-treated sperm are shown for the 3D reconstruction generated using RELION3 as reported previously 19. Gold-standard Fourier Shell Correlation (FSC) curve calculated between half maps of mouse sperm doublets. The resolution was estimated as 26 Å (FSC = 0.143). (D) Two slices of the 96 nm-repeating structure of doublets looking along and perpendicular to the filament axis. Note the red line in the top panel indicates the plane of the bottom slice and periodic structures are observed inside the microtubules. The coordinates were recentered on the 48-nm repeats and imported into RELION4. In the top panel, note the features further away from the microtubules are blurrier, suggesting that there are conformational heterogeneities and they are resolved at lower resolutions. (E) The initial Refine3D job of the 48-nm repeating structures was performed using RELION4 24. (F) The 3D reconstructions were matched to the 2D projections of individual particles in the raw tilt images and this step refined both the geometric and optical parameters of the tilt series. (G) Another round of subtomogram averaging was performed based on refined tilt series. No additional improvement was observed after 3 rounds of refinement and Refine3D as shown in (F)-(G). [file NIHMS1939567-supplement-9.tif]

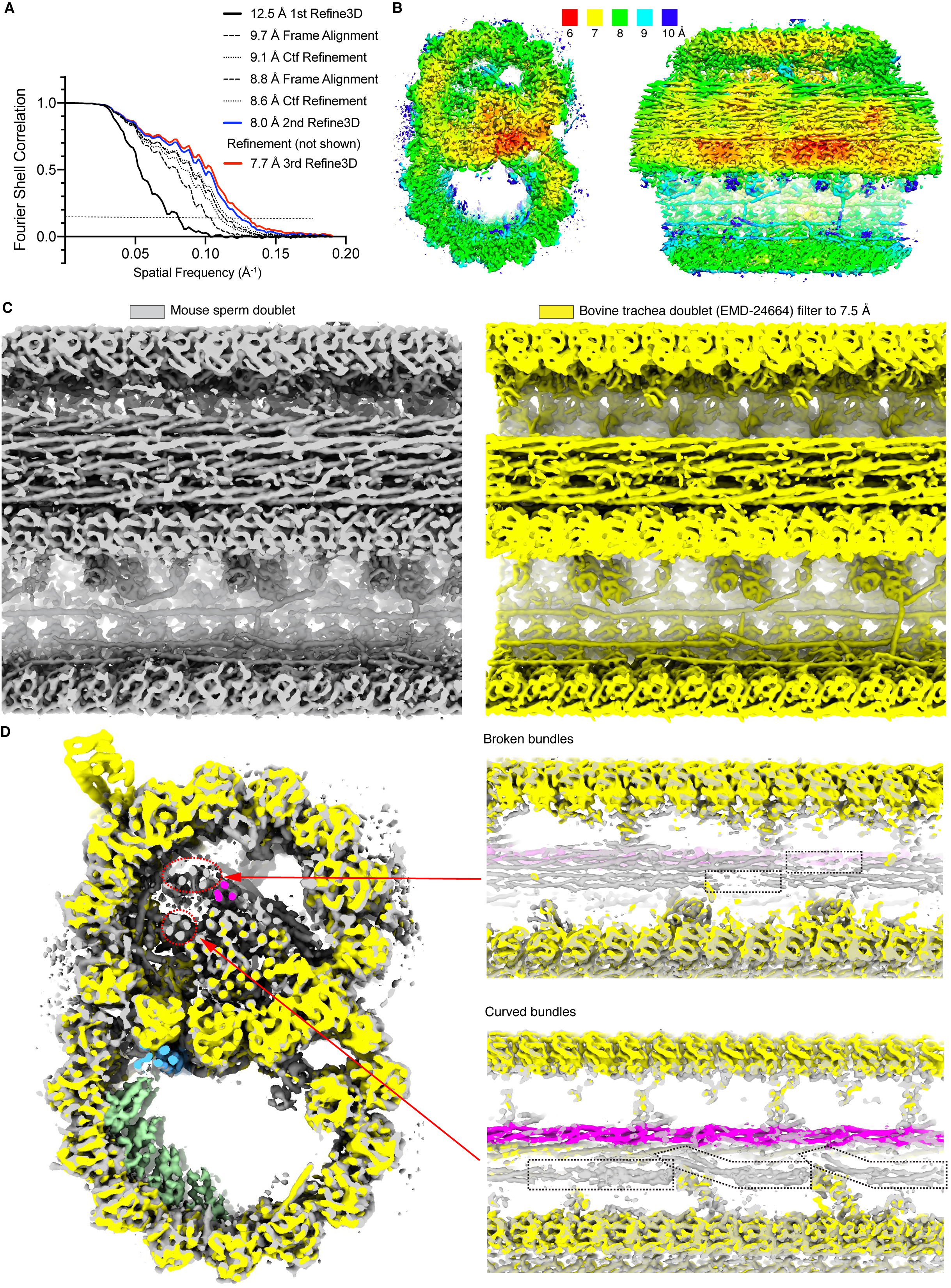

Supplement: 10 — Figure S2. Characterization of the 48 nm-repeating structure of doublets from mouse sperm. Related to Figures 1 and 3. (A) Gold-standard Fourier Shell Correlation (FSC) curves were calculated between half maps of mouse sperm doublets. The resolutions were reported as FSC = 0.143. Note the FSC curves resulting from the iterative frame alignment and CTF refinement between the second and third Refine3D jobs were not shown for the clarity of the figure. Further refinement after the third Refine3D did not improve the resolution or quality of the map. (B) The local-resolution map of mouse sperm doublets was calculated by RELION4. The ribbon region has the highest resolutions. Densities in the A-tubule have higher resolutions than the ones from the B-tubule. (C) Equivalent longitudinal cross-section views of doublets from mouse sperm and bovine trachea cilia (EMD-24664) are shown 13. The latter was low-pass filtered to 7.5 Å and comparable details of the secondary and tertiary structures of the MIPs are observed. (D) The reconstruction of mouse sperm doublet (grey) is overlaid with the bovine trachea doublets (yellow). The mouse sperm-specific densities are highlighted (dashed ovals). The broken helical bundles and the curved helical bundles inside the A-tubule of mouse sperm doublets along the microtubule axis are shown. The discontinuous parts of the broken helical bundles are indicated (dashed rectangles). Note the curved bundles have one straight and two curved groups of densities in every 48-nm repeat (outlined using dashed shapes). [file NIHMS1939567-supplement-10.tif]

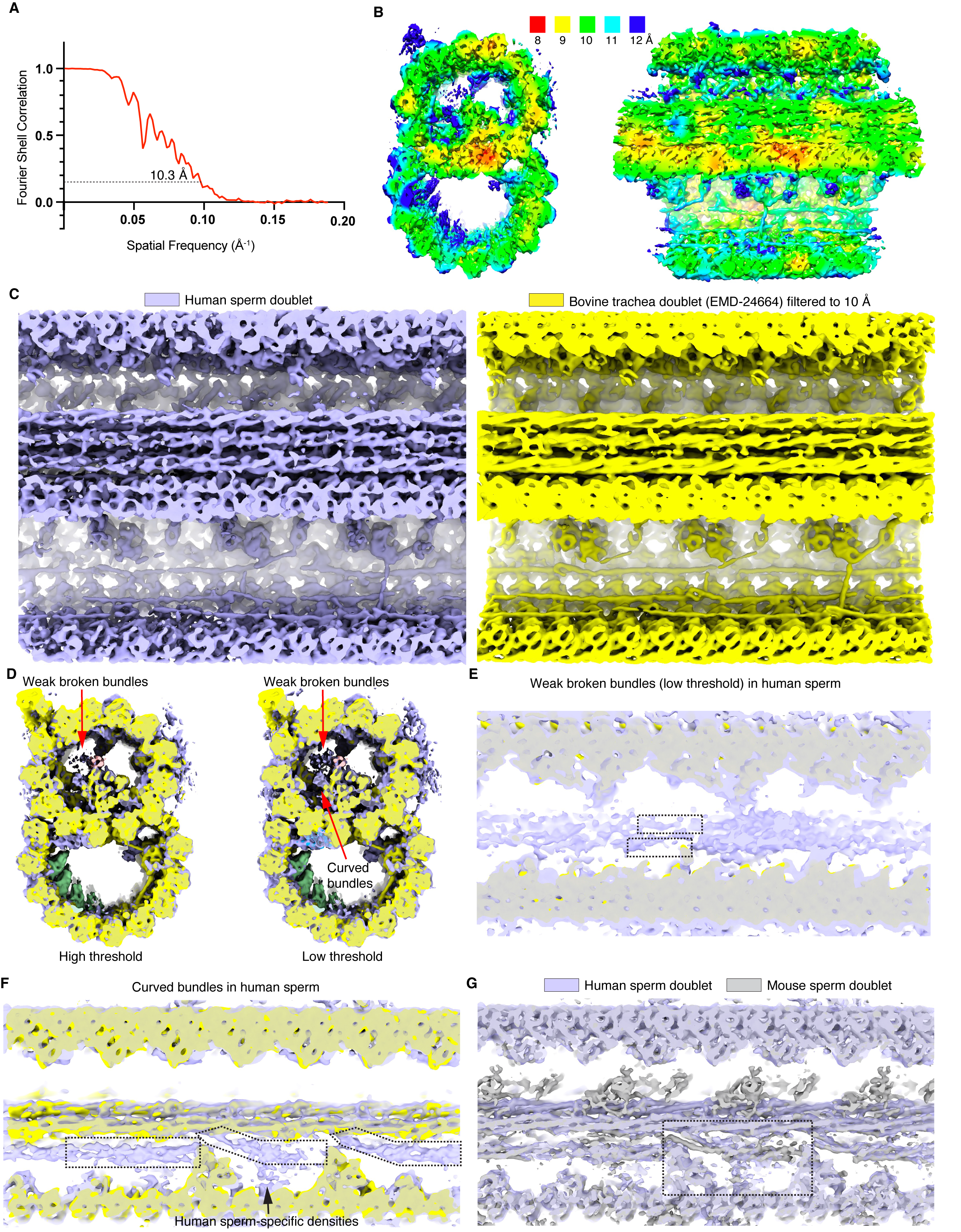

Supplement: 11 — Figure S3. Characterization of the 48 nm-repeating structure of doublets from human sperm. Related to Figure 1. (A) A gold-standard Fourier Shell Correlation (FSC) curve was calculated between half maps of mouse sperm doublets. The resolution was estimated as 10.3 Å (FSC = 0.143). (B) The local-resolution map of human sperm doublets was calculated by RELION4. The ribbon region has the highest resolutions. Densities in the A-tubule have higher resolutions than the ones from the B-tubule. (C) Equivalent views of doublets from human sperm and bovine trachea cilia (EMD-24664) are shown 13. The latter was low-pass filtered to 10 Å and comparable details of the secondary and tertiary structures of the MIPs are observed. (D) The reconstruction of human sperm doublet (blue) is overlaid with the bovine trachea doublets (yellow) at low and high thresholds. (E) The two broken bundles inside the A-tubule in human sperm are shown at a low threshold (see the corresponding mouse densities in Figure S2D). (F) The curved helical bundles contain one straight and two curved groups of densities inside the A-tubule of human sperm are outlined. Human sperm-specific densities were observed to connect one curved bundle to the lumen of A-tubule. (G) The human sperm doublets overlaid with mouse sperm doublets are shown. The inconsistent densities are outlined (dashed line) (also see Figures S2D and S3F). [file NIHMS1939567-supplement-11.tif]

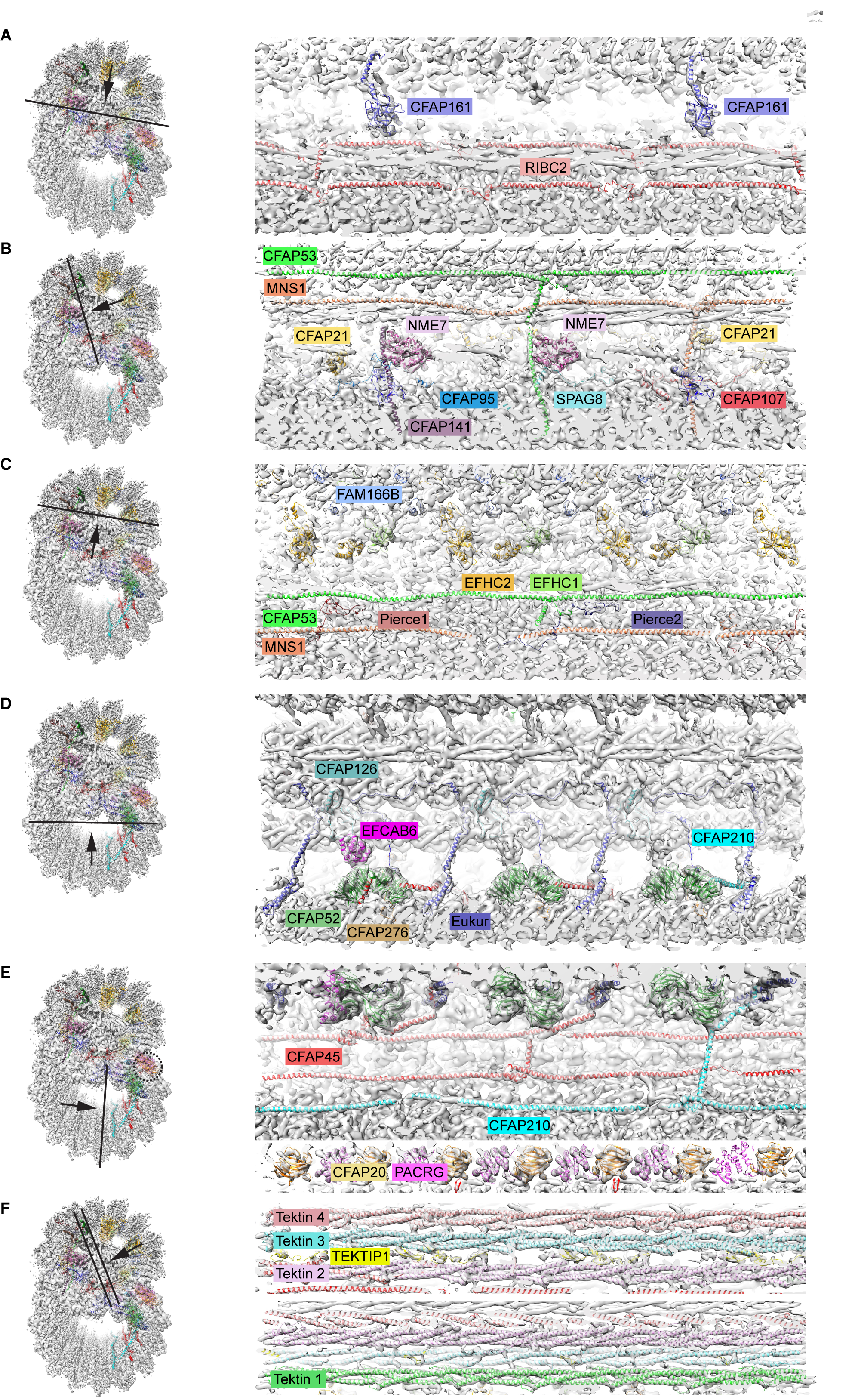

Supplement: 12 — Figure S4. Rigid-body fitting of 29 identified MIPs from bovine trachea cilia into the density map of mouse sperm doublet. Related to Figures 2, 3 and STAR Methods. (A)-(F), Models of 29 known MIPs from bovine trachea cilia (PDB 7RRO) 13 are fitted into the density map of mouse sperm doublet. The viewing angles for all panels are shown. For proteins that have multiple α-helices (CFAP161, RIBC2, CFAP53, MNS1, CFAP21, NME7, CFAP141, EFHC1, EFHC2, ENKUR, CFAP210, EFCAB6, CFAP45, PACRG and TEKTIN 1–4), the arrangement of secondary structures matches densities in sperm doublets. The overall shapes of β-sheet-rich proteins (CFAP52 and CFAP20) match the densities and these proteins are highly conserved in axonemes. For the proteins that contain random coils, we did observe matching features in the maps but it is generally harder to trace the main chains at the current resolution (CFAP95, SPAG8, CFAP107, FAM166B, Pierce1, Pierce2, CFAP126, CFAP276 and TEKTIP1). [file NIHMS1939567-supplement-12.tif]

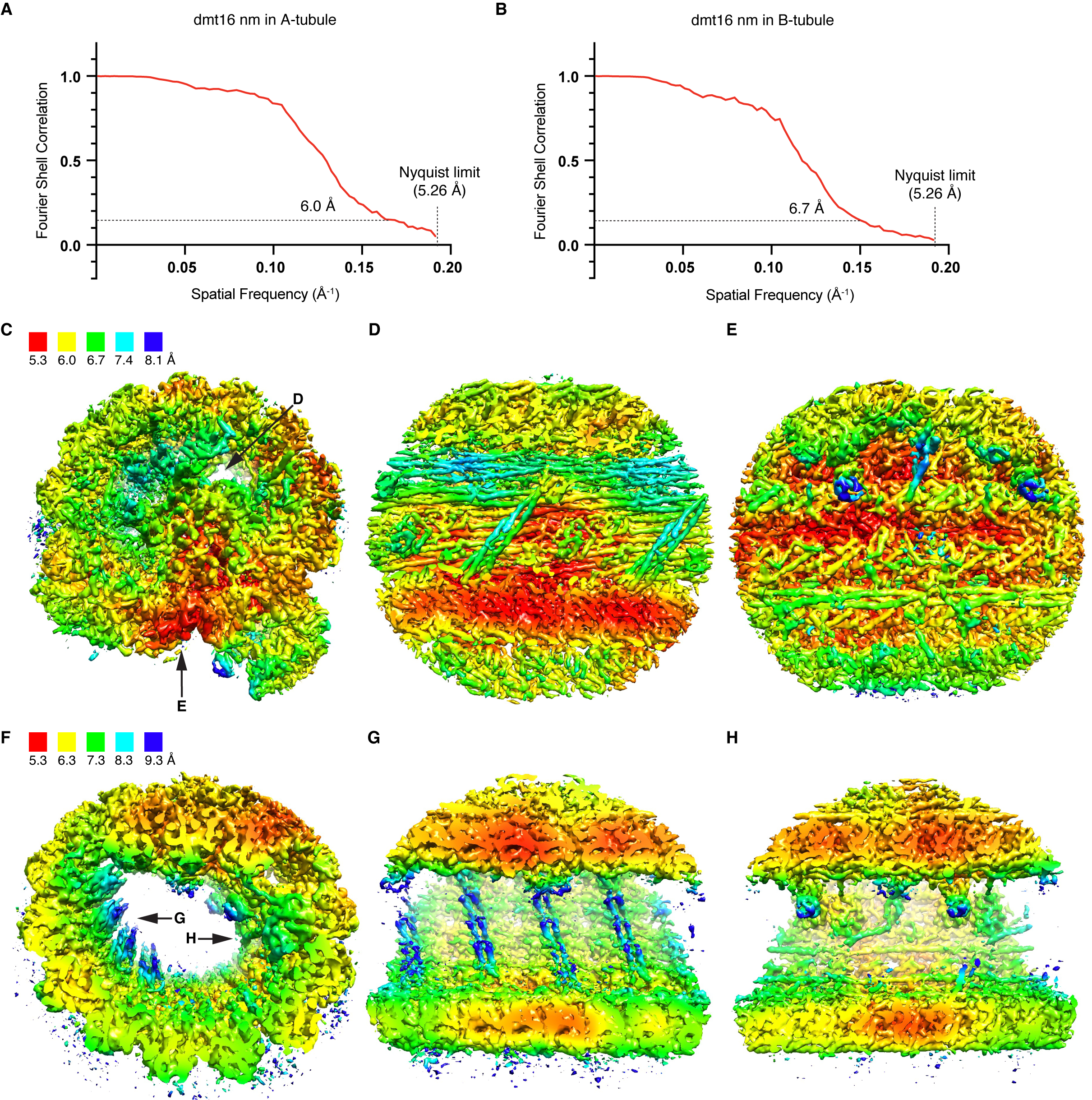

Supplement: 13 — Figure S5. Characterization of the 16 nm-repeating structures of doublets from mouse sperm. Related to Figure 2. (A)-(B) Gold-standard Fourier Shell Correlation (FSC) curves were calculated using half maps of 16 nm-repeating structures of A-tubule and B-tubule. The resolution was estimated as 6.0 Å and 6.7 Å, respectively (FSC = 0.143). The Nyquist limit is 5.30 Å. (C)-(E), The local resolution map was calculated from the two half maps of 16 nm-repeating structures of A-tubule using RELION4. The viewing angles for (D) and (E) are shown in (C) (black arrow). These viewing angles are similar to Figures 1A, 1D and 1E, respectively. (F)-(H), The local resolution map was calculated using half maps of 16 nm-repeating structures of B-tubule using RELION4. The viewing angles for (G) and (H) are shown in (F) (black arrow). The viewing angles of (F) and (G) are similar to Figure 1A and F, respectively. [file NIHMS1939567-supplement-13.tif]

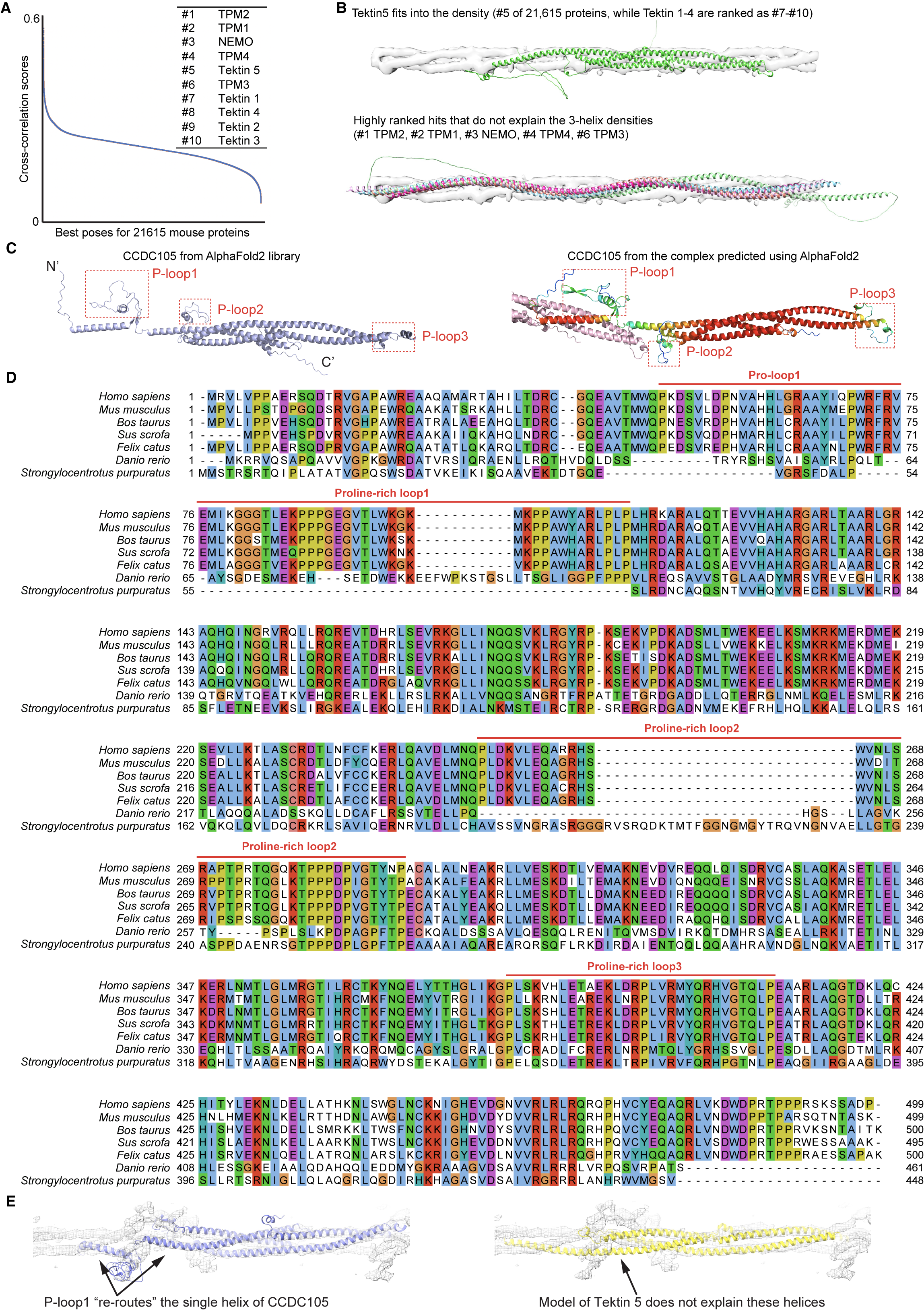

Supplement: 14 — Figure S6. Tektin 5 and CCDC105 likely form sperm-specific 3-helix bundles associated with the A-tubule. Related to Figures 2 and 3. (A) After unbiased matching, Tektin 5 was scored as the #5 hit of the predicted structures out of 21,615 proteins from the mouse proteome, ranked by cross-correlation scores (Top 10 are shown). Tektin 1–4 were ranked at #7–10 due to their similar tertiary structures. (B) Typical false positives (#1–4 and #6) from the same search. Usually, these are proteins with long single helices that do not match the gaps observed in the map. Also, they do not explain the 3-helix bundles. The fitting of Tektin 5 into the same densities is shown for comparison. (C) The structure of CCDC105 directly predicted by AlphaFold2 (left) is compared to the predicted complex formed by two CCDC105 molecules (right). The full-length CCDC105 molecule in the complex is colored based on the per-residue confidence scores (predicted local distance difference test, or pLDDT) from the AlphaFold2 prediction. The three P-loops have medium confidence scores (green), suggesting the exact conformations of these loops may not be accurately predicted. However, the presence of these structured loops is conceivably confident based on the conserved proline residues (see the sequence alignment in (D)) and matched the protrusion densities observed in our maps (Figure 2G). Note the conformations of the three proline-rich loops differ in these two predictions. These differences could be caused by the presence of neighboring molecules 27. (D) The sequence alignment of CCDC105 from five mammals (H. sapiens, M. musculus, B. taurus, S. scrofa and F. catus), zebrafish (D. rerio) and sea urchins (S. purpuratus). The three proline-rich loops are marked above the sequences. (E) The models of CCDC105 and Tektin 5 are fitted into the densities of the 3-helix bundle at the ribbon, where the former model explains the extra protrusions and orientation/lengths of helices of the densities but the [file NIHMS1939567-supplement-14.tif]

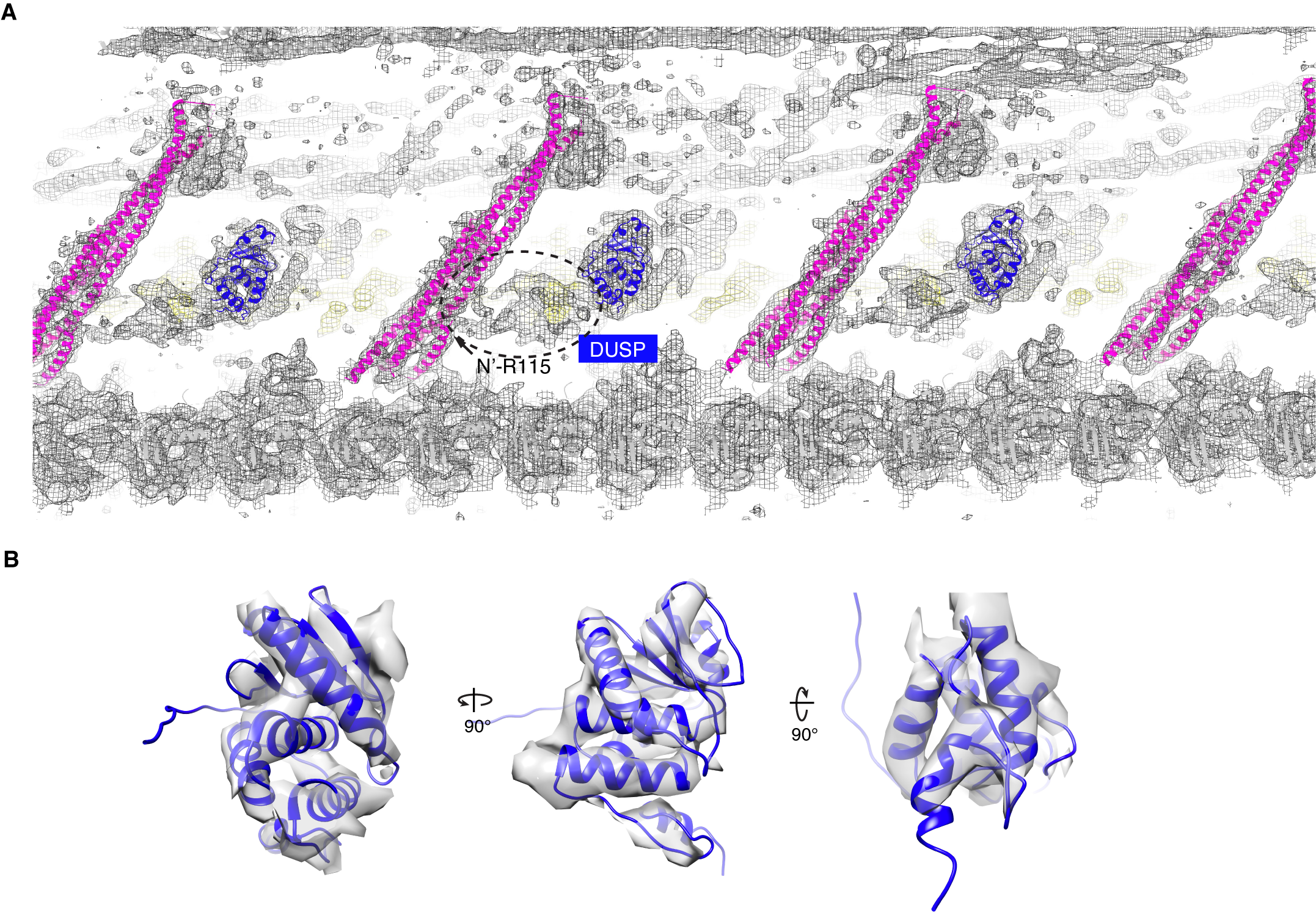

Supplement: 15 — Figure S7. DUSP proteins in the A-tubule. Related to Figure 3. (A) At a lower threshold compared to Figure 3C, densities connecting the N-terminal residues of the slanted Tektin 5s (magenta models) and the DUSPs (blue models) are observed. (B) The DUSP3 is fitted into the globular domain and three orthogonal views are shown. Other homologous DUSP proteins fit well into the density because of similar tertiary structures (DUSP 3, 13, 14, 18, 21 and 29). [file NIHMS1939567-supplement-15.tif]

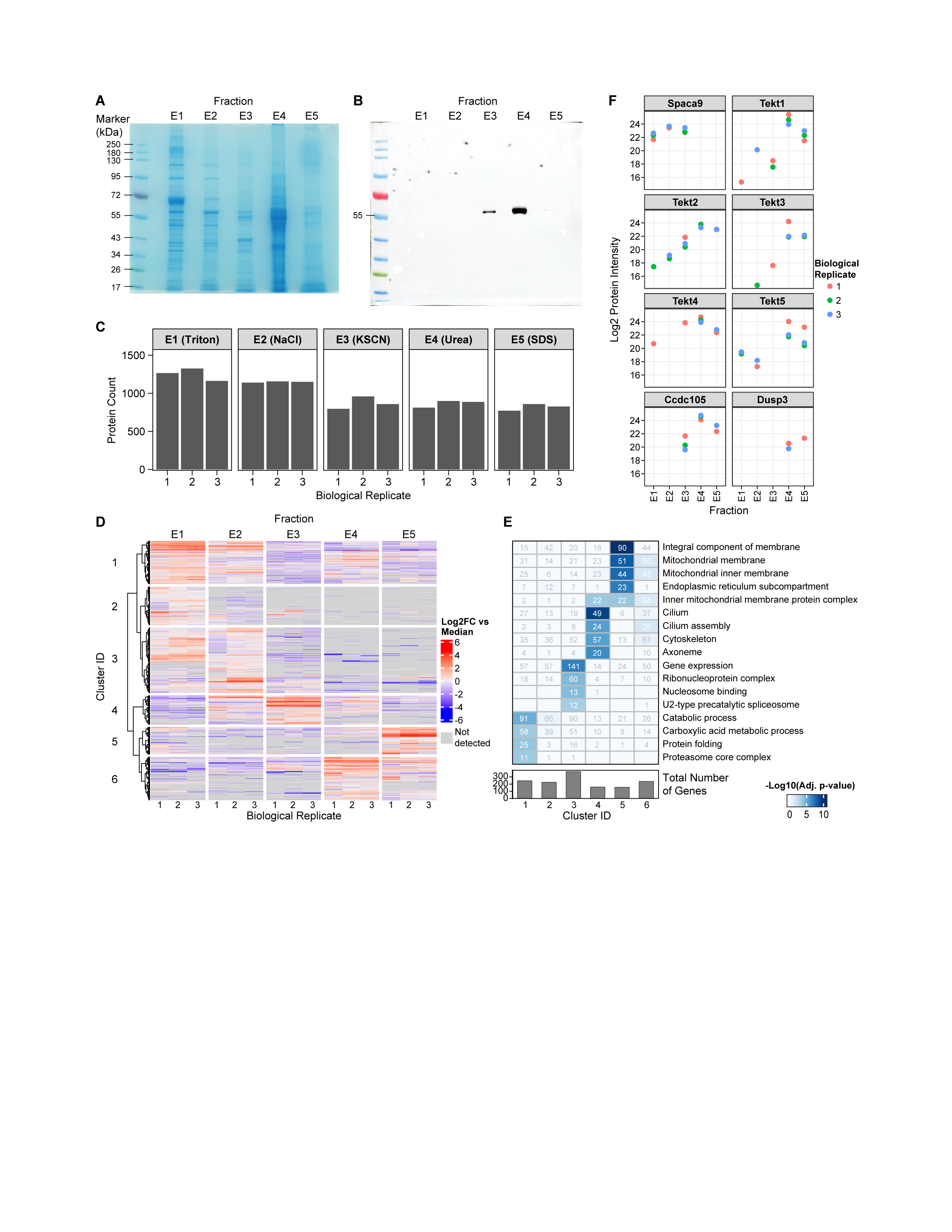

Supplement: 16 — Figure S8. Biochemical extractions of proteins from mouse sperm. Related to Figure 3. (A) SDS-PAGE analyses of protein extractions from mouse sperm using 0.1 % Triton in PBS (E1), 0.6 M NaCl in PBS (E2), 0.6 M KCSN in PBS (E3), 8 M urea (E4) and 10% SDS (E5). (B) Western blot analyses of protein extractions from mouse sperm using an antibody against α-tubulins. Note strong bands were detected only in E3 and E4, suggesting the microtubule structures were stable in Triton and high NaCl buffer, and dissembled completely in KCSN/urea solutions. (C) Bar chart of the number of proteins identified by MS (Protein Count) in each fraction (E1-E5) and biological replicate. We identified a total of 1,677 mouse proteins, with a range of 772 to 1,326 proteins identified in each individual fraction and replicate. (D) Heatmap of proteins with significant changes between any two fractions (absolute log2FC > 1, adjusted p-value < 0.05), listed by fractions (E1-E5) and biological replicate and clustered by correlation of intensity profile. Proteins are colored by the log2 fold change (log2FC) in protein intensity normalized to the row median (red, increased intensity; blue, decreased intensity; grey, not detected). Cluster identification numbers (Cluster ID) are labeled (left). (E) Heatmap of gene ontology (GO) enrichments among the significantly changing proteins identified in each cluster from (D) (left to right: Cluster ID 1–6, as labeled in D). GO terms were curated from the top 4 enrichment terms per cluster, and non-redundant terms were selected by an automated clustering procedure (see Materials and Methods). Increased shading reflects increased significance of the enrichment term. The number of proteins per enrichment term is shown in white if significant (adjusted p-value < 0.05), and grey if not significant (adjusted p-value > 0.05). A bar chart plotting the number of total genes in each cluster ID is included 48. (F), Log2 protein intensities (y-axis) for eight mouse protei [file NIHMS1939567-supplement-16.tif]
